# Genetic diversity of highly pathogenic avian influenza H5N6 and H5N8 viruses in poultry markets in Guangdong, China, 2020-2022

**DOI:** 10.1128/jvi.01145-24

**Published:** 2024-12-04

**Authors:** Kang Yang, Sarea Nizami, Shu Hu, Lirong Zou, Huishi Deng, Jiamin Xie, Qianfang Guo, Kimberly M. Edwards, Vijaykrishna Dhanasekaran, Hui-Ling Yen, Jie Wu

**Affiliations:** 1 Guangdong Provincial Center for Disease Control and Prevention 111659 , Guangzhou, Guangdong, China; 2 School of Public Health, Sun Yat-Sen University 26469 , Guangzhou, Guangdong, China; 3 School of Public Health, Li Ka Shing Faculty of Medicine, The University of Hong Kong 25809 , Hong Kong, Hong Kong; 4 HKU-Pasteur Research Pole, Li Ka Shing Faculty of Medicine, The University of Hong Kong 25809 , Hong Kong, Hong Kong; Emory University School of Medicine, Atlanta, Georgia, USA

**Keywords:** highly pathogenic avian influenza virus, clade 2.3.4.4, reassortment, surveillance, genetic diversity

## Abstract

H5 highly pathogenic avian influenza (HPAI) viruses of the A/Goose/Guangdong/1/96 (Gs/Gd) lineage continue to evolve and cause outbreaks in domestic poultry and wild birds, with sporadic spillover infections in mammals. The global spread of clade 2.3.4.4b viruses via migratory birds since 2020 has facilitated the introduction of novel reassortants to China, where avian influenza of various subtypes have been epizootic or enzootic among domestic birds. To determine the impact of clade 2.3.4.4b re-introduction on local HPAI dynamics, we analyzed the genetic diversity of H5N6 and H5N8 detected from monthly poultry market surveillance in Guangdong, China, between 2020 and 2022. Our findings reveal that H5N6 viruses clustered in clades 2.3.4.4b and 2.3.4.4h, while H5N8 viruses were exclusively clustered in clade 2.3.4.4b. After 2020, the re-introduced clade 2.3.4.4b viruses replaced the clade 2.3.4.4h viruses detected in 2020. The N6 genes were divided into two clusters, distinguished by an 11 amino acid deletion in the stalk region, while the N8 genes clustered with clade 2.3.4.4 H5N8 viruses circulating among wild birds. Genomic analysis identified 10 transient genotypes. H5N6, which was more prevalently detected, was also clustered into more genotypes than H5N8. Specifically, H5N6 isolates contained genes derived from HPAI H5Nx viruses and low pathogenic avian influenza in China, while the H5N8 isolates contained genes derived from HPAI A(H5N8) 2.3.4.4b and A(H5N1) 2.3.2.1c. No positive selection on amino acid residues associated with mammalian adaptation was found. Our results suggest expanded genetic diversity of H5Nx viruses in China since 2021 with increasing challenges for pandemic preparedness.

**IMPORTANCE:**

Since 2016/2017, clade 2.3.4.4b H5Nx viruses have spread via migratory birds to all continents except Oceania. Here, we evaluated the impact of the re-introduction of clade of 2.3.4.4b on highly pathogenic avian influenza (HPAI) virus genetic diversity in China. Twenty-two H5N6 and H5N8 HPAI isolated from monthly surveillance in two poultry markets in Guangdong between 2020 and 2022 were characterized. Our findings showed that clade 2.3.4.4h, detected in 2020, was replaced by clade 2.3.4.4b in 2021–2022. H5N6 (*n* = 18) were clustered into more genotypes than H5N8 (*n* = 4), suggesting that H5N6 may possess better replication fitness in poultry. Conversely, the H5N8 genotypes are largely derived from the clade 2.3.4.4b wild bird isolates. As clade 2.3.4.4b continues to spread via migratory birds, it is anticipated that the genetic diversity of H5N6 viruses circulating in China may continue to expand in the coming years. Continuous efforts in surveillance, genetic analysis, and risk assessment are therefore crucial for pandemic preparedness.

## INTRODUCTION

Outbreaks of highly pathogenic avian influenza (HPAI) viruses may cause high mortality in specific domestic poultry species and significant economic loss. Outbreaks of HPAI are often rapidly eliminated or eradicated through intervention, with only few viral lineages that continue evolving and causing outbreaks (1). One of them is the A/goose/Guangdong/1/96-like (Gs/Gd) lineage of H5Nx HPAI virus, which has evolved and persisted through diverse clades and genotypes since it first emerged in Southern China in 1996. These viruses affect not only domestic poultry but also spill over into wild birds and mammalian species. Historically, the H5 hemagglutinin (HA) gene of Gs/Gd H5 HPAI viruses has been most commonly associated with the N1 neuraminidase (NA) gene, forming the well-known H5N1 subtype. However, since 2014, genetic reassortment events have resulted in the emergence of other H5Nx subtypes, including H5N2, H5N5, H5N6, and H5N8 (2). As the first avian influenza strain documented to cause lethal human infections, the Gs/Gd-like H5N1, H5N6, and H5N8 viruses have led to over 900 human cases since 1997, primarily through contact with infected animals (3, 4). Since 2003, 896 H5N1 infections have been reported in 24 countries, with a case fatality at 52% (5). Since 2014, 93 cases of H5N6 infections have been reported from China and Laos (4), and H5N8 has been detected from asymptomatic poultry workers in Russia (6).

The HA gene of Gs/Gd-like viruses has evolved and diversified into multiple clades, with clade 2 and its various subclades gained global predominance after 2003. Spillover infections in migratory birds have facilitated the intercontinental spread of the Gs/Gd-like viruses, leading to poultry outbreaks in different regions of the world. Notable instances include clade 2.2 in 2005, clade 2.3.4.4c in 2014/15, and clade 2.3.4.4b since 2016/17 (7). The resurgence of clade 2.3.4.4b viruses in wild birds since 2020/2021 has driven a panzootic affecting both domestic poultry and wild birds globally (7). Clade 2.3.4.4 viruses are classified into eight subclades (a to h). All eight subclades have been detected in domestic poultry in China, with clades 2.3.4.4a, b, d, g, and h also caused human infections (8, 9). Since 2020, the predominant clade of H5N6 in China has shifted from clade 2.3.4.4h to clade 2.3.4.4b (8, 10).

In addition to the continued evolution of the HA gene, genetic reassortment of clade 2.3.4.4 viruses with local avian influenza viruses (AIVs) in different geographic regions has expanded genetic diversity over time, generating new genotypes and various H5Nx subtypes (11–14). Although Asia has been the epicenter for Gs/Gd-like viruses in the past, the current panzootic clade 2.3.4.4b viruses have undergone multiple genetic reassortment events, acquiring avian influenza gene segments derived from other regions of the world (15, 16). The re-introduction of the reassortant clade 2.3.4.4b viruses to China via migratory birds (15, 17) may influence the epidemiology and evolutionary dynamics of enzootic Gs/Gd-like viruses circulating in the region.

In this study, we conducted monthly surveillance at two poultry markets in Guangdong, China from 2020 to 2022. We performed genetic analysis on 22 HPAI H5Nx viruses isolated during this period. Our analyses focused on the genetic composition of these isolates, evaluating if gene segments introduced via migratory birds may affect the local AIV genetic diversity.

## RESULTS

### Detection of H5Nx HPAI viruses from poultry markets in Guangzhou, 2019–2022

**Fig 1:**
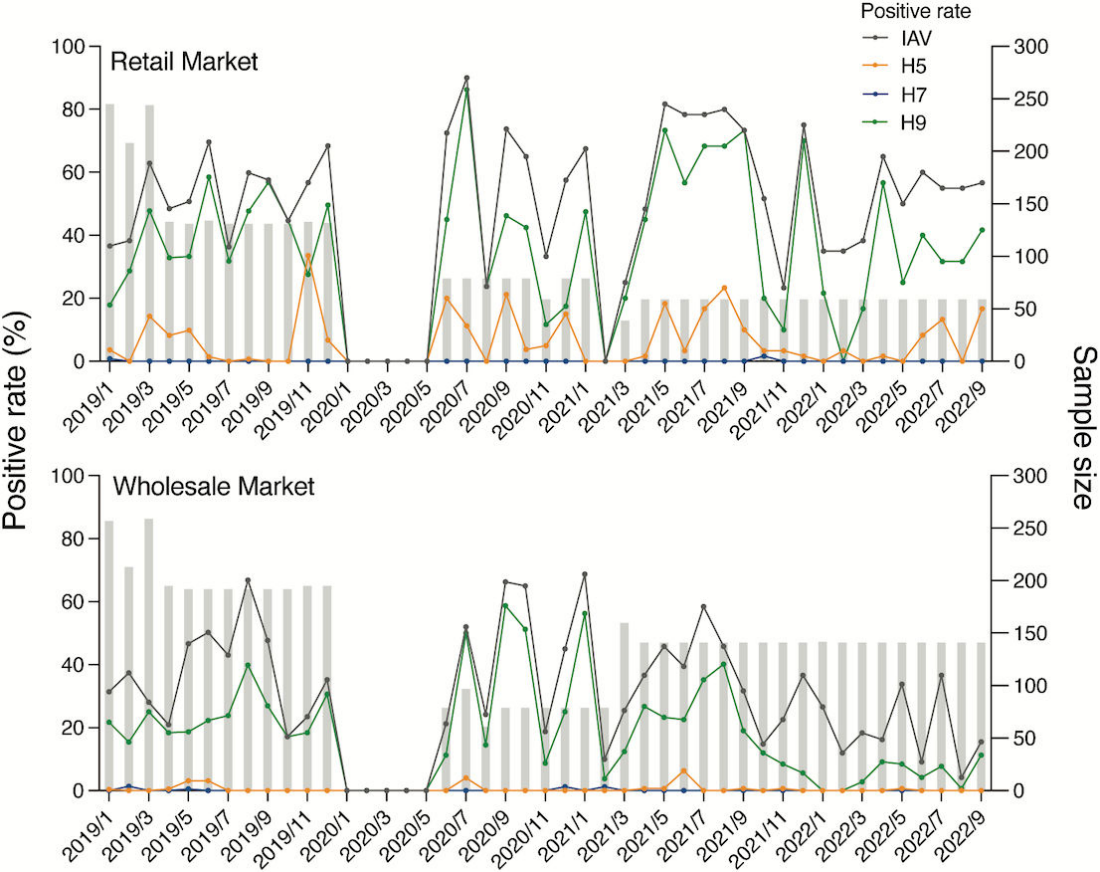
Monthly detection frequencies of H5, H7, and H9 AIV from retail and wholesale poultry markets in Guangdong between January 2019 and September 2022. Poultry swabs were collected every other week or monthly from a retail and a wholesale poultry market in Guangzhou, covering the period from January 2019 to September 2022, except for January to May 2020 (due to coronavirus disease-2019 lockdown) and February 2021 (retail market closure). AIV were detected by quantitative real-time RT-PCR targeting the influenza A virus M gene. AIV positive samples were further tested using primers and probes for H5, H7, and H9 subtypes by quantitative real-time RT-PCR. The number of swabs collected every month was shown in gray bars. The monthly detection frequencies of H5 (orange), H7 (blue), and H9 (green) from all sample types (oropharyngeal swabs, cloacal swabs, dressed poultry cavity, environmental swabs) were shown. Positivity rates by sample type are shown in Fig. S1.

Surveillance at two poultry markets has been ongoing since 2015, with results from 2015 to 2018 previously reported (18). From 2019 to 2022, AIV detection was more frequent in the retail market, with a median monthly positive rate of 56.7% (range 23.3%–90.0%), compared with 32.8% (range 4.2%–68.8%) in the wholesale market (*P* < 0.001, *t*-test) (Fig. 1). Among the routinely monitored H5, H7, and H9 subtypes, H9 was the most frequently detected, with median monthly positivity rates of 41.7% in the retail market and 18.4% in the wholesale market. H5 was predominantly detected in the retail market (range 0%–33.6%) and less prevalent in the wholesale market (range 0%–6.3%), while H7 detection remained low (<1.7%) in both markets (Fig. 1). Notably, oropharyngeal and environmental samples exhibited higher positivity rates than cloacal or cavity swabs in both markets (Fig. S1). Overall, H5 detection rates from 2020 to 2022 were comparable to those reported for 2015–2018 (18).

Of the 290 H5-positive samples collected during 2020–2022, 22 viruses were isolated (Table 1), comprising H5N6 (*n* = 18) and H5N8 (*n* = 4) viruses. Specifically, H5N6 viruses were detected in samples from chickens (*n* = 6), ducks (*n* = 5), and environmental sources (*n* = 7), while H5N8 viruses were detected from ducks (*n* = 3) and environmental samples (*n* = 1) (Table 1).

**TABLE 1:** Summary of H5Nx viruses isolated from poultry markets, Guangdong

Virus	Subtype (clade)	Sample type	Time of sample collection	Accession number	Genotype	NA stalk deletion
Wholesale market						
A/environment/Guangdong/MG169433/2020	H5N6 (2.3.4.4h)	Chopping board^*a*^	July 2020	EPI_ISL_19210563	G02	Yes
A/duck/Guangdong/KGZ15783/2021	H5N8 (2.3.4.4b)	Oropharyngeal	April 2021	EPI_ISL_19210571	G08	No
A/duck/Guangdong/KGZ15999/2021	H5N8 (2.3.4.4b)	Oropharyngeal	July 2021	EPI_ISL_19210556	G09	No
A/duck/Guangdong/KGZ16007/2021	H5N6 (2.3.4.4b)	Oropharyngeal	July 2021	EPI_ISL_19210557	G01	Yes
A/duck/Guangdong/KGZ16008/2021	H5N6 (2.3.4.4b)	Cloacal	July 2021	EPI_ISL_19210558	G01	Yes
A/duck/Guangdong/KGZ16009/2021	H5N6 (2.3.4.4b)	Oropharyngeal	July 2021	EPI_ISL_19210559	G01	Yes
A/duck/Guangdong/KGZ16011/2021	H5N6 (2.3.4.4b)	Oropharyngeal	July 2021	EPI_ISL_19210560	G01	Yes
A/duck/Guangdong/KGZ16012/2021	H5N6 (2.3.4.4b)	Cloacal swab	July 2021	EPI_ISL_19210561	G01	Yes
A/duck/Guangdong/KGZ16015/2021	H5N8 (2.3.4.4b)	Oropharyngeal	July 2021	EPI_ISL_19210562	G10	No
A/environment/Guangdong/MG171831/2021	H5N6 (2.3.4.4b)	Desk^*a*^	November 2021	EPI_ISL_19210565	G04	Yes
Retail market						
A/environment /Guangdong/MG169895/2020	H5N6 (2.3.4.4h)	Water^*a*^	September 2020	EPI_ISL_19210564	G03	Yes
A/chicken/Guangdong/MG170989/2021	H5N6 (2.3.4.4b)	Oropharyngeal	April 2021	EPI_ISL_19210543	G01	Yes
A/chicken/Guangdong/MG171127/2021	H5N6 (2.3.4.4b)	Dressed poultry cavity	May 2021	EPI_ISL_19210578	G01	Yes
A/chicken/Guangdong/MG171361/2021	H5N6 (2.3.4.4b)	Cloacal swab	July 2021	EPI_ISL_19210552	G01	Yes
A/chicken/Guangdong/MG171771/2021	H5N6 (2.3.4.4b)	Cloacal swab	October 2021	EPI_ISL_19210553	G04	Yes
A/environment/Guangdong/MG172783/2022	H5N8 (2.3.4.4b)	Chopping board^*a*^	June 2022	EPI_ISL_19210566	G10	No
A/environment/Guangdong/MG172933/2022	H5N6 (2.3.4.4b)	Chopping board^*a*^	July 2022	EPI_ISL_19210567	G05	No
A/chicken/Guangdong/MG173168/2022	H5N6 (2.3.4.4b)	Dressed poultry cavity	September 2022	EPI_ISL_19210554	G06	No
A/chicken/Guangdong/MG173169/2022	H5N6 (2.3.4.4b)	Dressed poultry cavity	September 2022	EPI_ISL_19210555	G07	No
A/environment/Guangdong/MG173172/2022	H5N6 (2.3.4.4b)	Desk^*a*^	September 2022	EPI_ISL_19210568	G07	No
A/ environment/Guangdong/MG173173/2022	H5N6 (2.3.4.4b)	Chopping board^*a*^	September 2022	EPI_ISL_19210569	G07	No
A/ environment/Guangdong/MG173174/2022	H5N6 (2.3.4.4b)	Knife^*a*^	September 2022	EPI_ISL_19210570	G07	No

^*a*^ Mixed poultry species were present in the markets; as such, the host origin of environmental samples is not available.

### Genetic analysis of the HA genes of H5Nx isolates

**Fig 2:**
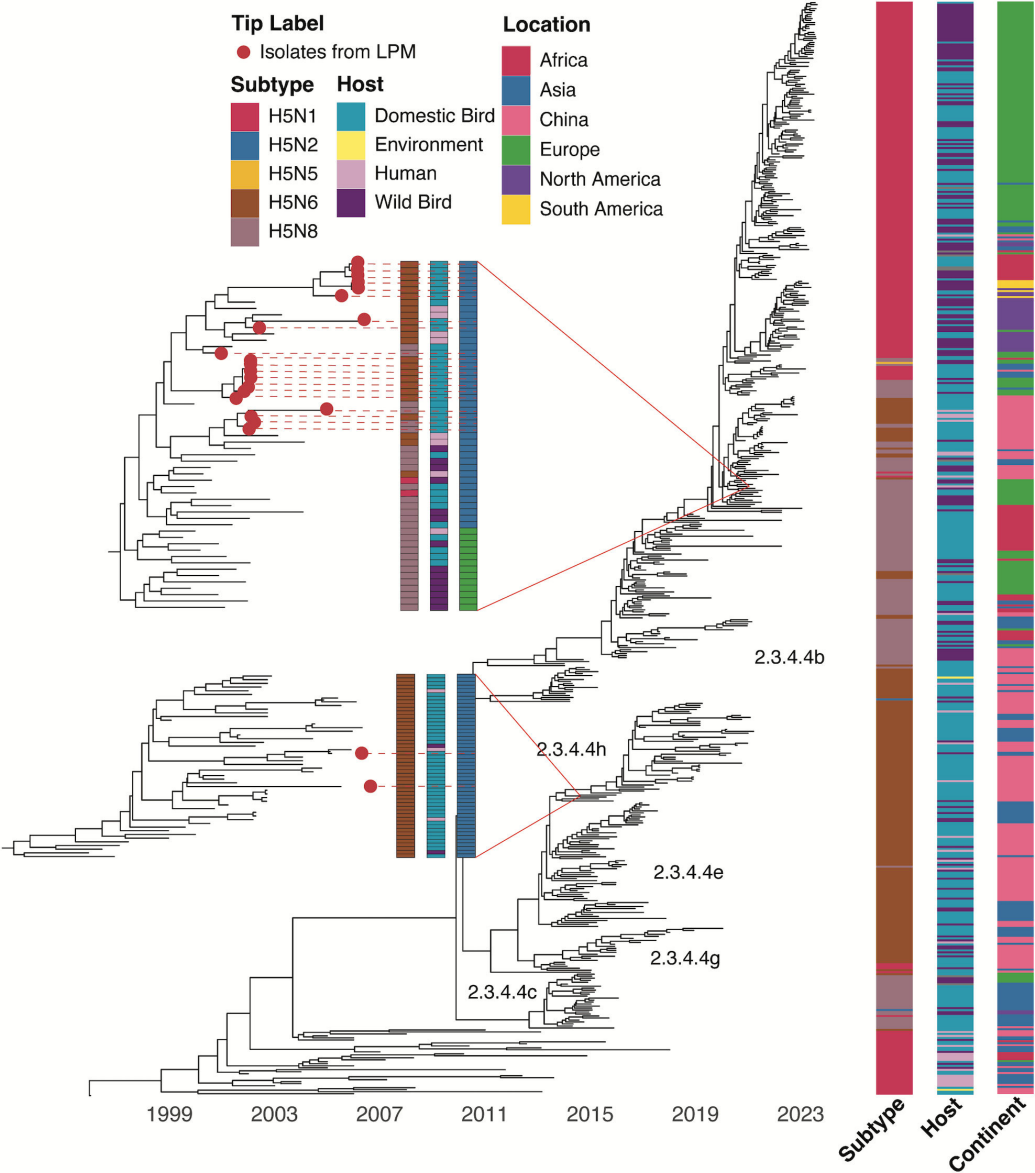
Genetic analysis of the HA gene of H5N6 and H5N8 viruses isolated from poultry markets in Guangdong, 2020–2022. Time-scaled maximum clade credibility tree of clade 2.3.4.4 HA genes (*n* = 550) detected globally from 01 January 2014 to 30 June 2023. Subtype, host, and geographic region (location) of the tips are shown as bars on the right. Figure key of the bars depicts all the categorical variables detected with respect to the figure. Red dots indicate the 22 H5N6 and H5N8 viruses isolated in this study.

Twenty-two H5N6 and H5N8 viruses isolated from poultry markets during 2020–2022 were sequenced for genetic analysis. All isolates were confirmed as HPAI by the presence of a polybasic amino acid sequence (PQRERRRKR/GLF or PQREKRRKR/GLF) in the H5 HA cleavage site (Table S1). A maximum clade credibility (MCC) HA tree was constructed with 550 selected sequences detected in 2014–2023 deposited in GISAID (Fig. 2) (19). The HA gene of the 22 HPAI H5Nx viruses isolated from poultry markets in Guangdong belonged to subclades 2.3.4.4h (*n* = 2) and 2.3.4.4b (*n* = 20). The endemic clade 2.3.4.4h viruses were only detected in 2020 and were replaced by the re-introduced clade 2.3.4.4b viruses in 2021–2022 (Table 1).

Clade 2.3.4.4h is primarily composed of H5N6 viruses isolated from domestic birds in China and other parts of Asia (Fig. 2; Fig. S2A). The two clade 2.3.4.4h H5N6 isolates from the poultry markets in Guangdong clustered closely with other H5N6 viruses circulating among chickens and ducks in Asia since 2015. The HA phylogeny shows that they are genetically related to A/duck/China/FJ19332/2019 and A/chicken/Shandong/2.25_TAWL001-O/2019 isolated in China in 2019 (Fig. S2A). One of these isolates, A/environment/Guangdong/MG169433/2020, shared 99.6% nucleotide similarity in HA gene with the human H5N6 isolate A/Anhui/2021-00011/2020 (Fig. S2A).

The HA genes of 20 clade 2.3.4.4b isolates of H5N6 and H5N8 subtypes were clustered with H5N8 viruses re-introduced into China since 2020 (15, 17), which have originated in Northern Africa and spread across Eurasia (20, 21) (Fig. 2; Fig. S2B). HA phylogeny shows that the 20 isolates were closely related to H5N6 and H5N8 viruses isolated from wild birds and domestic birds in China and Vietnam in 2020 and 2021, as well as to H5N6 viruses isolated from humans in China in 2021 and 2022 (Fig. S2B). The mean time of most recent common ancestor (tMRCA) of clade 2.3.4.4b viruses detected in Guangdong was in June 2020 (95% higher posterior density, March 2020 to August 2020). Our results suggest that the re-introduced clade 2.3.4.4b has become enzootic among domestic poultry in Guangdong.

### Genetic analysis of the N6 and N8 genes of the H5Nx isolates

**Fig 3:**
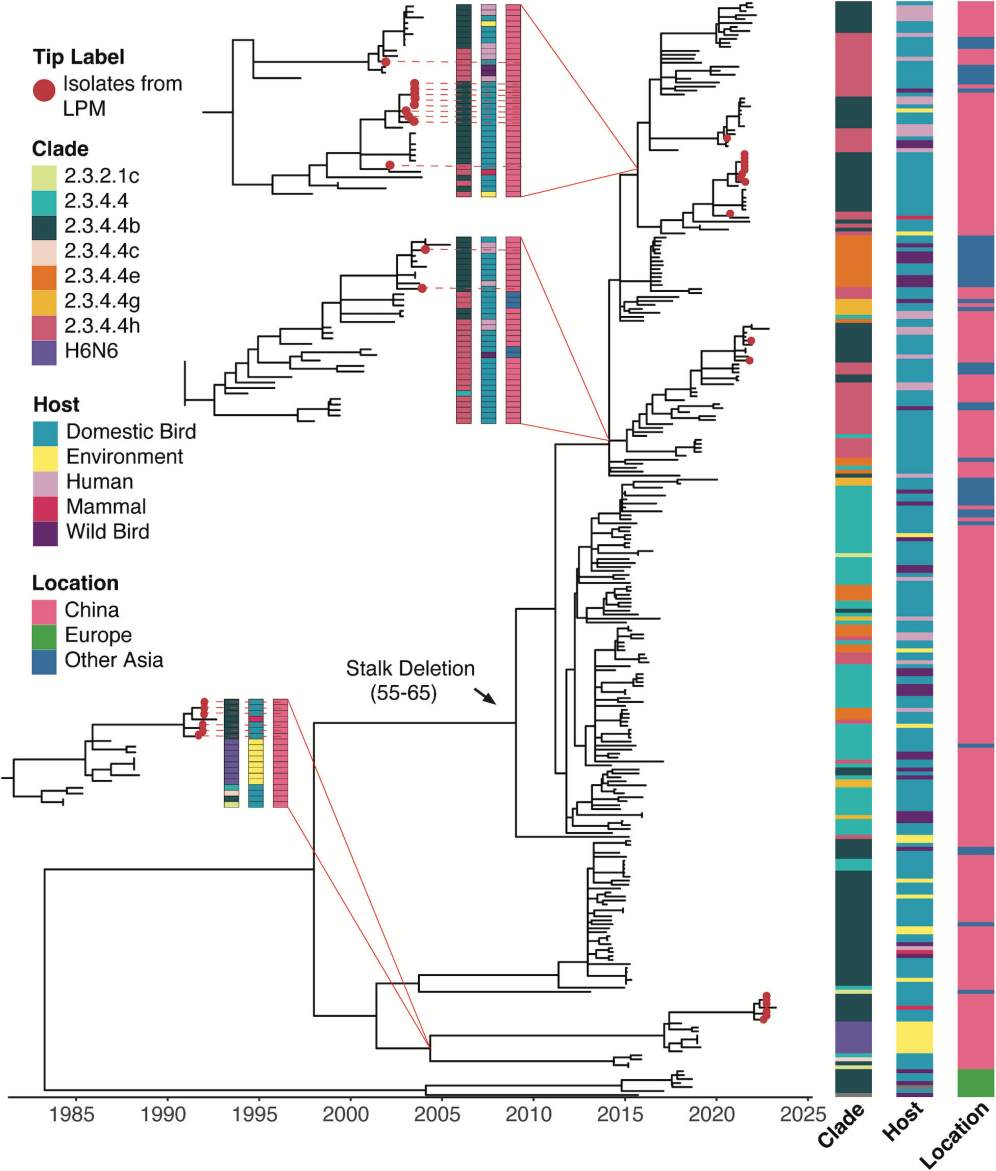
Genetic analysis of the N6 genes of H5N6 viruses isolated from poultry markets in Guangdong, 2020–2022. Time-scaled maximum-likelihood tree of N6 genes of A(H5N6) viruses (*n* = 276) detected globally from 01 January 2014 to 30 June 2023. HA clade information, host, and geographic region (location) of the tips are shown as bars on the right. Figure key of the bars depicts all the categorical variables detected with respect to the figure. Red dots indicate the 18 H5N6 viruses isolated in this study.

**Fig 4:**
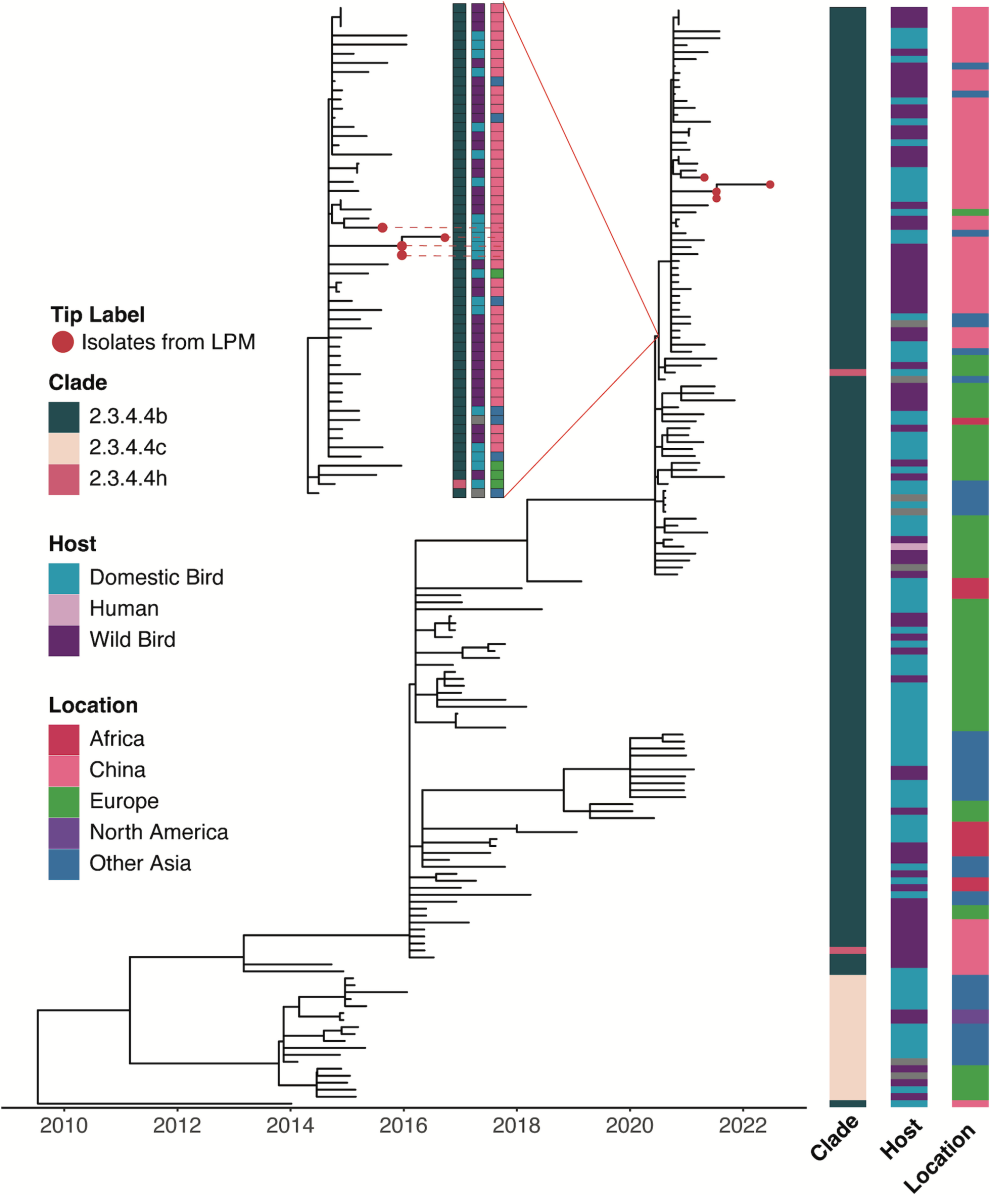
Genetic analysis of the N8 genes of H5N8 viruses isolated from poultry markets in Guangdong, 2020–2022. Time-scaled maximum-likelihood tree of N8 genes of A(H5N8) viruses (*n* = 158) detected globally from 01 January 2014 to 30 June 2023. HA clade information, host, and geographic region (location) of the tips are shown as bars on the right. Figure key of the bars depicts all the categorical variables detected with respect to the figure. Red dots indicate the four H5N8 viruses isolated in this study.

Among the 22 H5Nx viruses isolated from the poultry markets, 4 were H5N8 and 18 were H5N6 viruses. Phylogenetic trees for the N6 and N8 genes were constructed using 276 and 158 selected sequences, respectively, from 2014 to 2023 deposited in GISAID (Fig. 3 and 4). These sequences were subsampled from the H5Nx sequences used for constructing the HA tree.

The N6 NA sequences (*n* = 18) were divided into two major clades. The larger clade (with *n* = 12 poultry market sequences) was further subdivided into multiple co-evolving clusters, containing mainly H5N6 viruses from domestic chickens and ducks in China (Fig. 3; Fig. S3). This N6 NA clade featured an 11 amino acid deletion in the stalk region (positions 55–65, N1 numbering) and paired with multiple 2.3.4.4 HA subclades (e.g., 2.3.4.4b, e, g, h) (Fig. 3). Among the 12 H5N6 viruses isolated from the poultry markets which contained a deletion in the N6 NA gene, 2 of them paired with HA from clade 2.3.4.4h while the remaining 10 paired with HA from clade 2.3.4.4b (Fig. 3; Fig. S3A). NA phylogeny shows the poultry market isolates were most closely related to clade 2.3.4.4h and 2.3.4.4b H5N6 viruses isolated from domestic poultry and humans in China in 2020 and 2021 (Fig. S3A). The smaller clade contained six clade 2.3.4.4b poultry market isolates with a full-length NA (Fig. 3; Fig. S3B). NA phylogeny shows these viruses were most closely related to clade 2.3.4.4b H5N6 viruses and low pathogenic AIV of H6N6 subtype isolated in China after 2018 (Fig. S3B). Since the N6 genes of H5N6 and low pathogenic H6N6 viruses were clustered, the results suggest genetic reassortment may have occurred between clade 2.3.4.4b and local AIV in domestic poultry.

The four clade 2.3.4.4b H5N8 isolates from the poultry markets shared 99.4%–100% nucleotide homology in the NA sequence and were closely clustered together (Fig. 4; Fig. S4). Related N8 gene segments were mainly identified from both wild and domestic birds from China since 2020 that share ancestry with wild and domestic birds in Europe and were mainly paired with clade 2.3.4.4b HA genes (Fig. S4).

### Genetic analysis on the internal gene segments of H5Nx viruses

**Fig 5:**
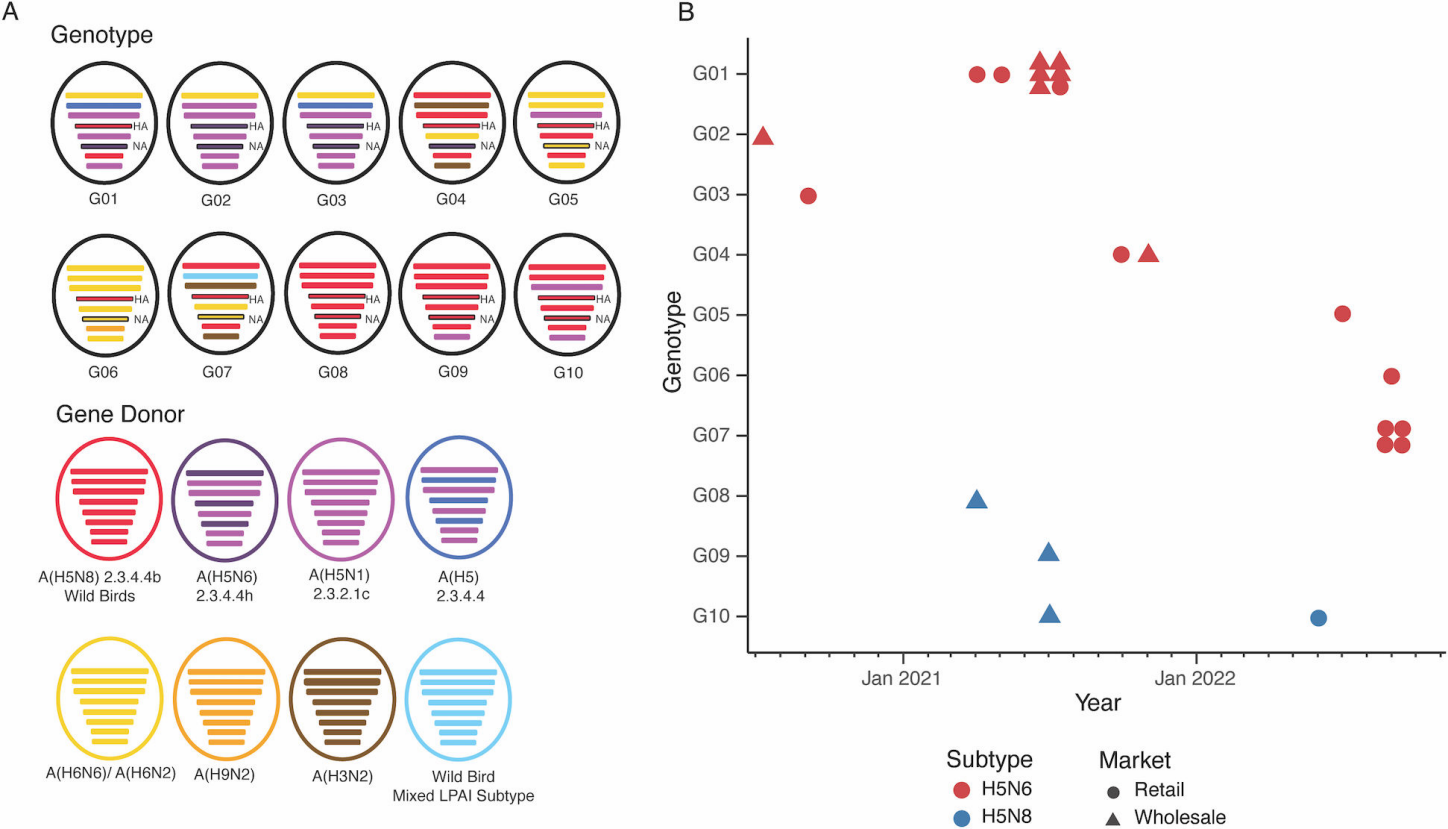
Genotype analysis of the 22 H5N6 and H5N8 viruses isolated from poultry markets in Guangdong, 2020–2022. (**A**) Ten genotypes were determined through identifying cluster combination using genotype progenitor strains in the phylogenetic trees (see Fig. S5 to S10). The representative strains of the gene pools are as follows: A(H5N8) 2.3.4.4b wild birds, A/Astrakhan/3212/2020(H5N8); A(H5N6) 2.3.4.4h, A/Goose/Guangdong/PO1707260055/GZH/2017(H5N6); A(H5N1) 2.3.2.1c, A/Anhui/1/2005(H5N1); A(H5Nx) 2.3.4.4, A/duck/Zhejiang/6DK19/2013(H5N2); A(H3N2), A/chicken/Ganzhou/GZ43/2016(H3N2); A(H9N2), A/Chicken/Zhejiang/HJ/2007(H9N2); and wild bird low pathogenic gene pool A/Eastern Spot-billed_Duck/Shanghai/JDS19701/2019(H4N2). The representative strains for the A(H6N6)/ A(H6N2) gene pool were A/duck/Ganzhou/GZ151/2016(H6N6), A/chicken/China/GZ1063/2014(H6N6), and A/duck/Guangdong/3111/2018(H6N2). (**B**) H5Nx viruses detected from the retail or the wholesale markets are shown as circles and triangles, respectively, according to genotype and date of sample collection.

Full genome analysis identified 10 genotypes from the 22 HPAI H5Nx isolates (Fig. 5A; Table 1; Fig. S5 to S10). Most genotypes were transient, detected only within a single month of sampling (Fig. 5B). Among the 10 genotypes, G01 to G07 belonged to the H5N6 subtype, while G08 to G10 were of the H5N8 subtype. Several genotypes, such as G01, G04, and G10 were detected at both the retail and wholesale markets, while the remaining genotypes were detected at either the retail or the wholesale markets. G01 was the most frequently detected genotype (*n* = 8), which was detected from both retail and wholesale markets in the span of 2 months (May 2021 to July 2021). G10 was the genotype with the longest circulation time, as it was detected twice in July 2021 and June 2022, respectively. Genotypes G02 and G03 were similar to the genotypes that have been previously identified in other studies (8). Specifically, both G02 and G03 differ only by the PB1 gene from the previously published genotype 2015A [A/Guangxi/13486/2017(H5N6)], which has been associated with human infections since 2015 (8). G04 is identical to the published genotype G7 A/duck/Hunan/S40199/2021(H5N6)] detected from domestic ducks and geese (22).

Among the 22 isolates, H5N6 (*n* = 18) were classified into seven genotypes (G01 to G07) while the H5N8 (*n* = 4) were classified into three genotypes (G08 to G10) (Fig. 5A). The G01 to G07 H5N6 genotypes contained genes derived from other Gs/Gd-lineage H5Nx viruses [e.g., A(H5N8) 2.3.4.4b, A(H5N6) 2.3.4.4h, A(H5N1) 2.3.2.1c, A(H5Nx) 2.3.4.4], low pathogenic AIVs from domestic poultry [e.g., A(H3N2), A(H6N6)/A(H6N2), A(H9N2)], and various subtypes of low pathogenic AIVs from wild birds (H4, H10, H11, and H12). Meanwhile, the three H5N8 genotypes (G8, G9, and G10) mainly contained genes derived from HPAI A(H5N8) 2.3.4.4b wild bird isolates and from A(H5N1) 2.3.2.1c. Although seven genotypes were identified for the H5N6 isolates, some of the genotypes share common gene segments. For example, genotypes G01, G02, and G03 shared common ancestry in five gene segments (PB2, PA, NP, NA, and NS). In addition, G01 to G05 possessed the N6 gene with an 11 amino acid stalk deletion, while G06 and G07 possessed N6 genes without stalk deletions (Fig. 3; Fig. S3B).

Among the 10 genotypes, substitution of the PB1 segment was the most frequently observed with six donor strains [e.g., A(H5N8) 2.3.4.4b, A(H5N1) 2.3.2.1c, A(H5Nx) 2.3.4.4, A(H3N2), A(H6N6)/A(H6N2), and wild bird gene pool] (Fig. S6). In comparison, substitution of the PB2 was the least frequently observed with only two donor strains [e.g., A(H5N8) 2.3.4.4b and A(H6N6)/A(H6N2)] (Fig. S5). Taken together, H5N6 was detected more frequently and formed more genotypes than the H5N8 viruses at the poultry markets in Guangdong from 2020 to 2022. The higher detection frequency of H5N6 suggests that they may be more adapted to domestic poultry, which provided an opportunity for genetic reassortment between HPAI and low pathogenic AIVs in the domestic poultry. Meanwhile, the H5N8 genotypes are genetically related with HPAI A(H5N8) 2.3.4.4b wild bird isolates with some genes derived from A(H5N1) 2.3.2.1c.

### Monitoring amino acid changes with host adaptation in H5Nx viruses

The 22 H5Nx isolates were evaluated for the presence of amino acid residues associated with increased virulence, mammalian adaptation (including transmissibility), and antiviral drug resistance (8, 23–26) (Table S1). In the HA gene segment, S137A, S158N, and T160A, known to increase binding to human-type receptors, were found among all the isolates (22/22; 100.00%). These mutations are similar to those reported in other H5N6 avian influenza viruses detected in China from 2020 to 2021 and in European avian isolates since October 2022 (22, 24, 27). Additionally, the HA mutations T192I (19/22; 86.36%) and S227L/R (21/22; 95.45%), associated with changes in receptor specificity and reported in H5N6 human infections, were also identified among the majority of the isolates (8). The HA adaptive mutations S137A, S158N, T192I, and S227l/R have been tested in the H5N1 subtype while S158N has been tested to decrease mice virulence in the H9N2 subtype (26). In the M1 gene, N30D, P41A, and T215A, along with L103F and L106M in the NS gene segment, were identified in all isolates (22/22; 100.00%). These mutations are associated with increased virulence and transmission (8). The M1 adaptive mutations N30D and T215A have been tested to increase mice virulence in the H5N1 subtype, while NS L106M mutation was tested to increase viral replication in mammalian cells virulence in mice through the H1N1 recombinant virus with internal genes from H7N9 (26). Notably, emerging mammalian-adaptive mutations reported in recent clade 2.3.4.4b H5N1 outbreaks in Europe, including PB2-T271A, NP-Y52N, and NA-S369I, were not observed in the 22 isolates from the poultry markets in Guangdong (0/22; 0.00%) (24, 28).

## DISCUSSION

The global dissemination of clade 2.3.4.4b H5Nx viruses via migratory birds has facilitated genetic reassortment between Gs/Gd-lineage HPAI viruses and regional low pathogenic AIVs, resulting in expanded genetic diversity of clade 2.3.4.4b viruses. Currently, the zoonotic potential of these genetically diverse clade 2.3.4.4b viruses remains uncertain, but genetic reassortment presents opportunities for generating influenza viruses with increased viral fitness. Understanding the genetic diversity of HPAI H5Nx viruses circulating in domestic poultry is crucial for pandemic preparedness, given the predominant association of zoonotic infections with poultry exposure. In the present study, we characterized 22 H5N6 (*n* = 18) and H5N8 (*n* = 4) HPAI viruses isolated from poultry markets in Guangdong between 2020 and 2022. While the H5N6 was clustered in both 2.3.4.4h and 2.3.4.4b, the H5N8 viruses were exclusively clustered with 2.3.4.4b viruses. Initially, the enzootic clade 2.3.4.4h was detected until September 2020, after which the re-introduced clade 2.3.4.4b has become predominant in H5N6 and H5N8 viruses detected from the poultry markets in 2021–2022. Notably, H5N6 viruses were detected more frequently and clustered into more genotypes than H5N8 viruses, suggesting an ongoing advantage for N6 NA in domestic poultry in China.

Deletion in the NA stalk region has been reported to correlate with AIV adaptation to land-based poultry (29, 30), although it is not required for HPAI viruses to contain a deletion in the NA gene (31). The N6 gene of H5N6 viruses was clustered into two distinct clades with and without an 11 amino acid deletion in the stalk region. The H5N6 viruses with the NA stalk deletion mainly circulated among domestic poultry in China since 2014, clustering in a clade with the NA genes paired with HA genes of multiple 2.3.4.4 subclades (e.g., 2.3.4.4b, e, g, h). In comparison, the H5N6 viruses with a full-length NA were mainly paired with HA of the 2.3.4.4b subclade. As the full-length N6 was also genetically related to low pathogenic H6N6 viruses detected in China after 2018, the results suggest genetic reassortment may have occurred between clade 2.3.4.4b and local low pathogenic AIV in domestic poultry. Further studies are needed to assess the replication fitness of clade 2.3.4.4b H5N6 viruses with different NA stalk lengths in different poultry species.

A total of 10 genotypes were identified among the 22 H5N6 and H5N8 viruses isolated from the poultry market. While H5 was detected more frequently in the retail market, where a higher degree of human-poultry-environment interaction is expected, the number of genotypes detected in the retail market (*n* = 7) was comparable with those detected in the wholesale market (*n* = 6) (Table 1). Additionally, H5N6 isolates that were more prevalently detected at the poultry markets were clustered into more genotypes than the H5N8 isolates. While our analysis cannot infer gene diffusion patterns, H5N6 isolates contained genes derived from the Gs/Gd-lineage H5Nx viruses and low pathogenic AIVs in China, suggesting they may have undergone reassortment in domestic poultry. Contrastingly, H5N8 isolates contained genes largely derived from the HPAI clade 2.3.4.4b A(H5N8) wild bird isolates with a few genes derived from A(H5N1) 2.3.2.1c. Most of the genotypes detected in this study were transient; however, this study is limited in the scope, sample size, and sampling frequency to infer persistence of genotypes across Southern China. To facilitate pandemic risk assessment of clade 2.3.4.4b viruses circulating in different regions of the world, there is a critical need to harmonize genotyping methodologies using robust computational approaches.

Human infection with the Gs/Gd-lineage H5N6 viruses has been predominantly documented predominantly from China since 2014, with 93 laboratory confirmed cases and a 40% case fatality rate (4). None of the 18 H5N6 isolates in our study shared genotypes with known human isolates from China. However, the HA and NA gene sequences are closely related to viruses that have caused human infections (Fig. 2 and 3). It is anticipated that the genetic diversity of H5N6 viruses circulating in China will continue to expand, facilitated by migratory birds introducing novel gene segments of clade 2.3.4.4b viruses from other regions of the world. Continuous efforts in surveillance, genetic analysis, and risk assessment analysis are essential for effective pandemic preparedness.

## MATERIALS AND METHODS

### Poultry market surveillance

Poultry swabs were collected every other week or monthly from a retail and a wholesale poultry market in Guangzhou, covering the period from January 2019 to September 2022, except for January to May 2020 (due to coronavirus disease-2019 lockdown) and February 2021 (retail market closure). Oropharyngeal swabs (2,973) and cloacal swabs (2,960) were obtained from live or dressed poultry, while an additional 1,068 swabs were collected from the cavity of dressed poultry. Concurrently, 2,551 environmental samples including air, fecal droppings, drinking water, poultry diet, wastewater, and swabs from surfaces involved in poultry processing (chopping board, knife, desktop) or poultry housing (cages), were collected. All samples underwent RT-PCR testing for influenza A virus detection using primers and probes targeting the M gene segment. Positive samples were further tested for H5, H7, or H9 subtypes using subtype-specific primers and probes (18).

### Virus isolation and next generation sequencing

Samples tested positive for H5 by RT-PCR with Ct values <35 were inoculated into the allantoic cavity of 9–11-day-old-specific pathogen free embryonated eggs (Jinan SPAFAS Poultry Company, Ltd.) and incubated for 24–48 hours at 37°C. Viral RNA was extracted from the harvested allantoic fluid using an automatic platform (BioGerm) and amplified with the One-step RT-PCR kit (Qiagen) and specific primers (5′-GGGGGGAGCRAAAGCAGG-3′ and 5′-CGGGTTATTAGTAGAAACAAGG-3′). For library preparation, Nextera XT library Prep kit (Illumina) was used, followed by sequencing on Illumina MiSeq. FASTAQ files were analyzed with CLC Genomics Workbench v20.0.4 (Qiagen) via *de novo* assembly. Contigs were generated using BLAST analysis against the NCBI GenBank influenza virus database, with the reference sequence displaying the highest percent matching identity selected for mapping to each segment for each isolate. The sequences of the isolates were deposited in GISAID (19) with the accession numbers listed in Table 1.

### Sequence data curation and phylogenetic analysis

HPAI H5Nx virus sequences and metadata from avian and human hosts (01 January 2014 and 30 June 2023) were obtained from GISAID on 19 July 2023 (*N* = 16,781). Avian isolates were categorized into wild or domestic species. The host classification system was refined using the host facet of the virus strain names. Species labeled as avian or other avian were marked as N/A. Classification limitations included insufficient information for determining outdoor access for poultry species and captivity status for wild species. Poultry was assumed as domestic origin unless specified in the metadata. Duplicate and mixed-subtype isolates were excluded based on isolate name. Sequences that diverged from a root-to-tip regression of sample collection dates and branch lengths from a preliminary maximum-likelihood phylogenetic tree constructed using FastTree v2.1.11 (32), utilizing Tempest v1.5.3, were considered erroneous and removed from subsequent analysis (33). H5 sequences representing published WHO candidate vaccine viruses were incorporated (34).

Multiple sequence alignments were conducted with MAFFT v7.490 (35, 36), and alignments were manually trimmed in AliView v1.27 (37). A maximum-likelihood phylogenetic tree was constructed using FastTree v2.1.11 (32) for clade assignment and to infer lineages introduced into China. Sequences of only clade 2.3.4.4 and clade 2.3.4.4a–h were retained (*N* = 12,956) for further analysis. Sequences of 99.5% nucleotide similarity were clustered by CD-HIT (38) to reduce sequence redundancy. A random sample proportion of 0.3 using Seqkit (39) was performed to reduce the data set. Subsequently, manual subsampling was performed to reduce the sequences in the tree using metadata information of host and continent (40).

The evolutionary history of the HA gene and divergence times were estimated using a Bayesian Markov chain Monte Carlo (MCMC) approach in BEAST v1.10.4 (41), enhanced with the BEAGLE high-performance library (42). We employed an uncorrelated relaxed molecular clock model to account for rate heterogeneity across branches, combined with a general time reversible nucleotide substitution model with gamma-distributed rate heterogeneity among sites. A Gaussian Markov Random Field Skyride coalescent model was used to capture fluctuations in population over time. A minimum of two separate MCMC runs were executed for each lineage, each consisting of 70 million states, with samples taken every 10,000 steps and a 20% burn-in. Results for each lineage were merged to ensure an effective sample size exceeding 200, verified in Tracer v1.7.1. The MCC tree was subsequently generated using TreeAnnotator v1.10.4 and annotated in R v4.2.2 (43) using packages “ggtree” v3.6.2 (44) and “ggplot2” v3.4.2 (45). To improve divergence estimates of the re-introduced lineages in China, we used BLAST v2.14.0+ (46) to identify the 10 most similar non-China sequences in GISAID for each sequence in the data set. After removing duplicate sequences, 88 contextual HA sequences were selected to be added with a percent identity similarity of 99.65%–100%.

For the NA and internal genes, BLAST v2.14.0+ (46) was used to identify the 10 most similar sequences in GISAID for each sequence from the poultry market in Guangdong, which were integrated alongside the study isolates for comprehensive analysis. Sequences of representative H5 strains previously published as lineage defining were included in these analyses (8, 10, 13, 15, 22, 47–57), and the trees were used to define genetically related groups (Fig S5 to S10). A gene that clustered with a specific genotype defining strain with a greater than 90% bootstrap value was considered to be genetically related and used for genotype definition . Time-dependent NA phylogenetic trees were constructed with IQ-TREE v2.2.0.7 using the least square dating method with 1,000 replicates while maximum-likelihood tree was generated by IQ-TREE v2.2.0.7 ultrafast bootstrap with 1,000 replicates (40). Phylogenies were annotated in R v4.2.2 (43) using packages “ggtree” v3.6.2 (44) and “ggplot2” v3.4.2 (45). All accession numbers used in the final phylogenies are listed in Table S2. The final data set size of HA, N6 NA, N8 NA, PB2, PB1, PA, NP, MP, and NS are 550, 276, 158, 1,114, 1,109, 1,109, 1,127, 1,128 and 1,119, respectively.

### Monitoring amino acid changes associated with host adaptation

Molecular markers associated with host adaptation or virulence were identified from the literature (8, 26). The HA and NA gene segments were numbered according to A/Aichi/2/1968(H3N2) (GISAID accession numbers EPI381955 and EPI381957) (58), while the internal gene segments were numbered according to A/Goose/Guangdong/1/1996(H5N1) (GenBank accession numbers AF144300 to AF144303 and AF144306 to AF144307).

## Data Availability

The sequences of 22 poultry market isolates were deposited in GISAID with the accession numbers listed in Table 1. Data, code, and analysis files for phylogenetic analysis are available at HKU Data Repository (doi:10.25442/hku.27312111).

## SUPPLEMENTAL MATERIAL

Supplemental material jvi.01145-24-s0001.docx Figures S1 to S10; Tables S1 and S2.

ASM does not own the copyrights to Supplemental Material that may be linked to, or accessed through, an article. The authors have granted ASM a non-exclusive, world-wide license to publish the Supplemental Material files. Please contact the corresponding author directly for reuse.
