# Supplementary material for: Genetic diversity of highly pathogenic avian influenza H5N6 and H5N8 viruses in poultry markets in Guangdong, China, 2020-2022
Source: J Virol. 2024 Dec 4;99(1):e01145-24. doi: 10.1128/jvi.01145-24 (PMC11784294; doi:10.1128/jvi.01145-24)
Supplement: Supplemental material — Figures S1 to S10; Tables S1 and S2. [file jvi.01145-24-s0001.docx]

**SUPPLEMENTARY MATERIALS**

**Supplementary Fig. 1.** Monthly positivity rates of H5, H7, and H9 avian influenza virus (AIV) subtypes detected from (A) oropharyngeal swabs, (B) cloacal swabs, (C) cavity swabs, and (D) environmental swabs in a retail and a wholesale market in Guangdong between January 2019 and September 2022. Poultry swabs were collected every other week or monthly from a retail and a wholesale poultry market in Guangzhou, covering the period from January 2019 to September 2022, except for January to May 2020 (due to COVID-19 lock-down) and February 2021 (retail market closure).


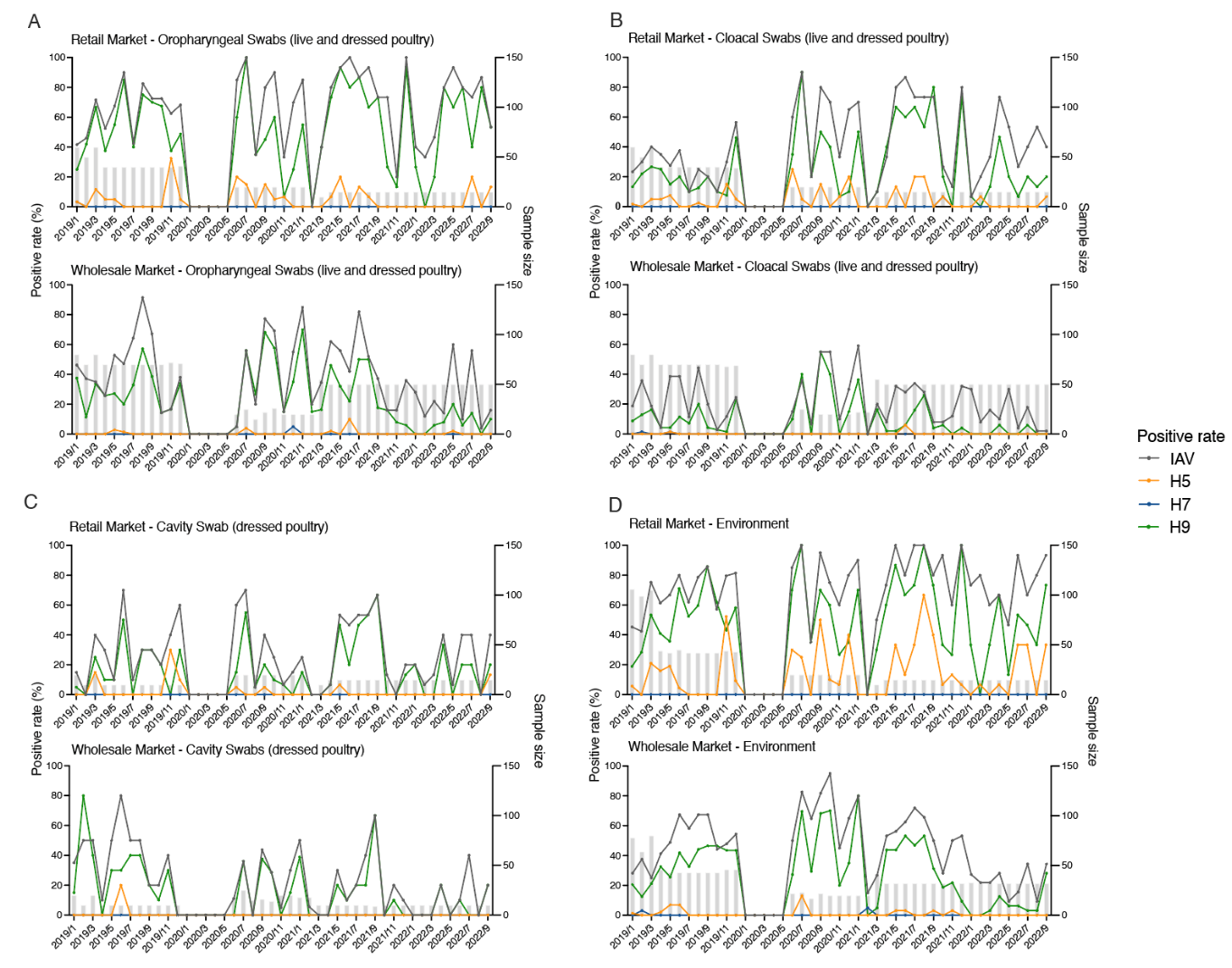


**Supplementary Fig. 2**. **H5 HA phylogenetic tree.** Expanded branches of (A) clade 2.3.4.4h and (B) clade 2.3.4.4b from the maximum clade credibility HA tree (Figure 2). Poultry market isolates were labeled in red text; human isolates were labeled in blue text. Scale bars indicate nucleotide substitutions per site. Subtype, host and geographic region (continent) of the tips.


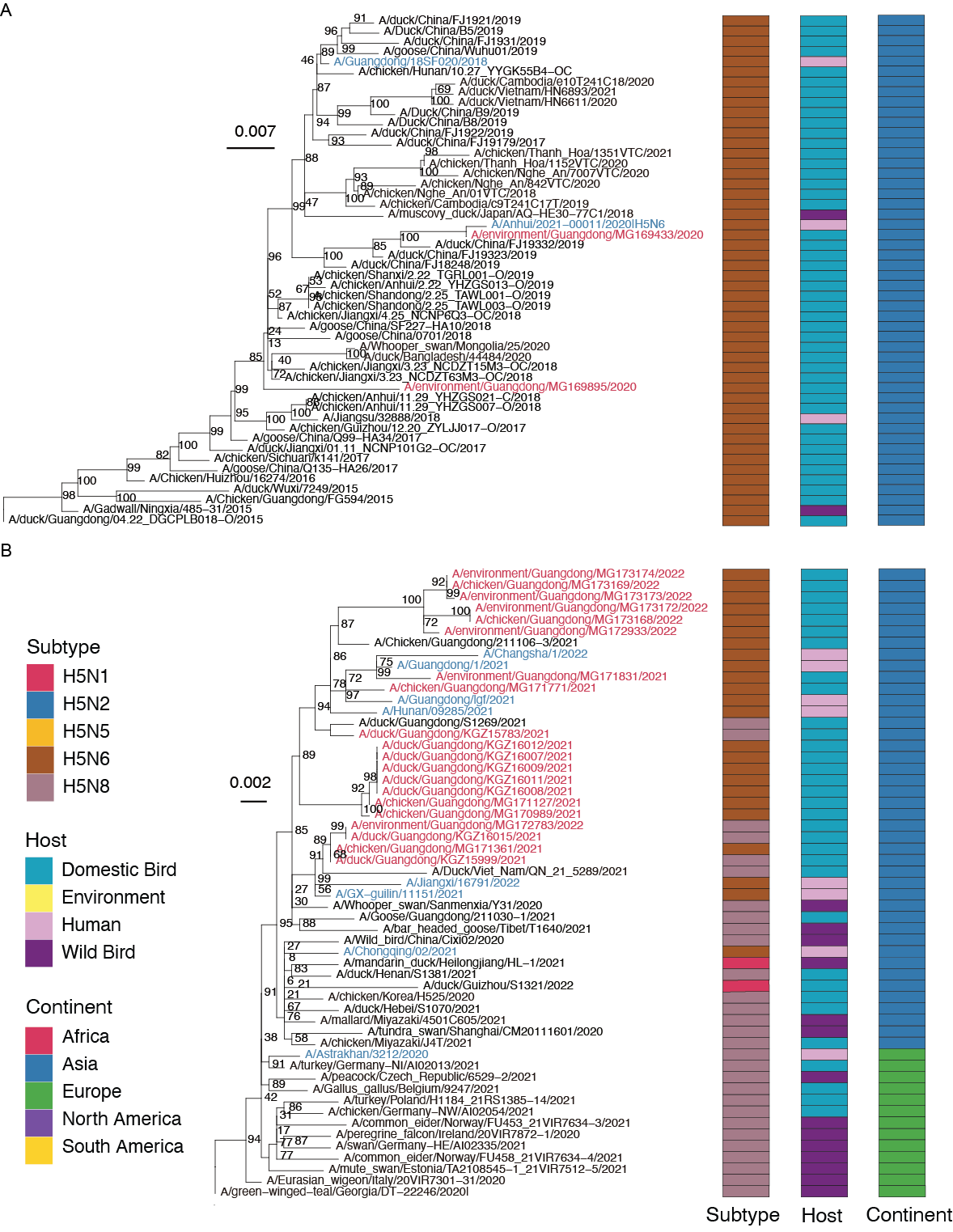


**Supplementary Fig. 3**. **Expanded branches of N6 phylogenetic tree.** Expanded branches of (A) 12 H5N6 isolates with NA stalk deletion and (B) 6 H5N6 isolates without NA stalk deletion from the maximum likelihood N6 tree (Figure 3). Poultry market isolates were labeled in red text. Scale bars indicate nucleotide substitutions per site. Clade, host and geographic region (continent) of the tips are shown as bars on the right.


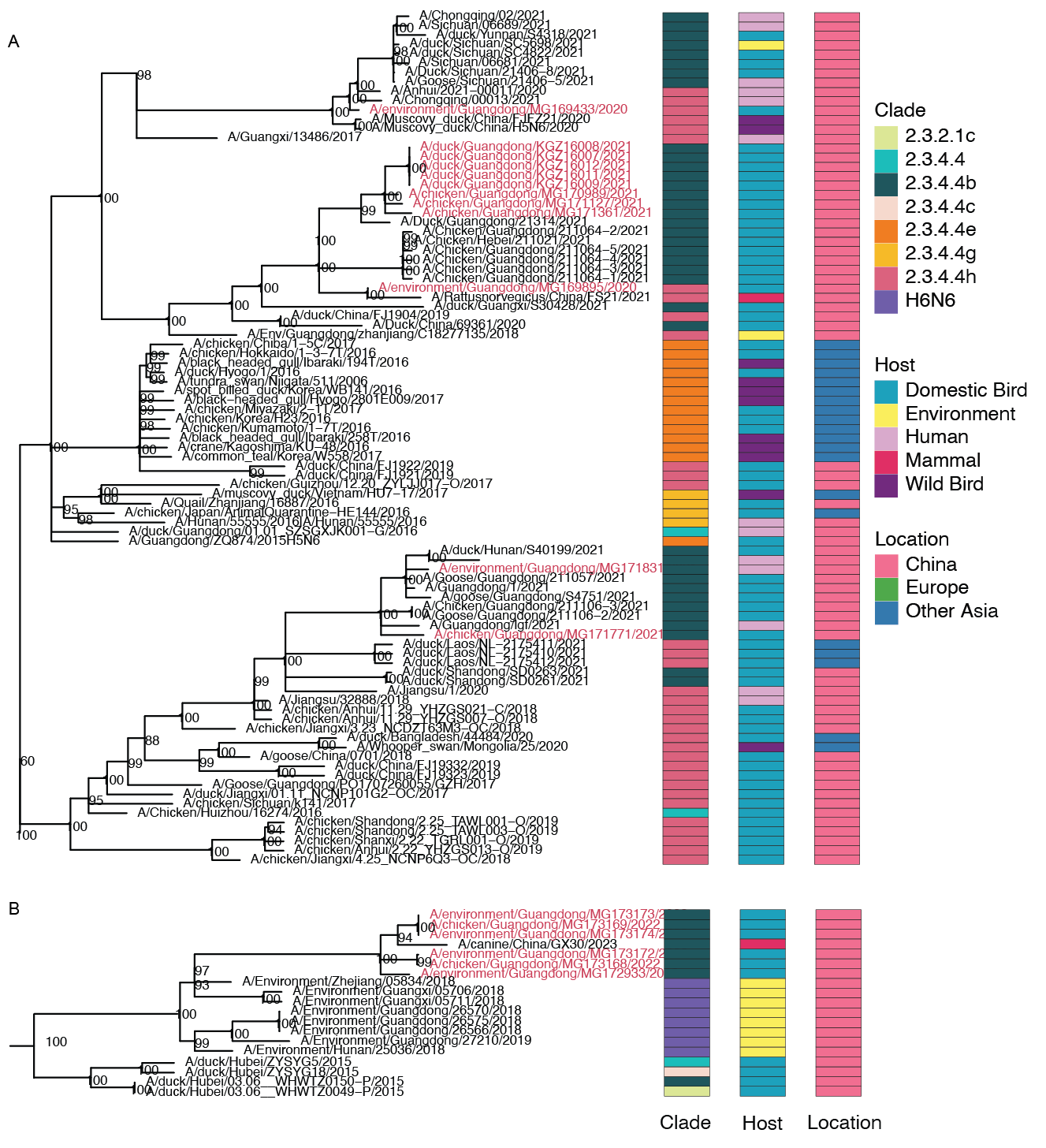


**Supplementary Fig. 4**. **Expanded branches of** **N8 phylogenetic tree.** Expanded branches of 4 H5N8 isolates from the maximum likelihood N8 tree (Figure 4). Scale bars indicate nucleotide substitutions per site. Poultry market isolates were labeled in red. Clade, host and geographic region (continent) of the tips are shown as bars on the right.

**
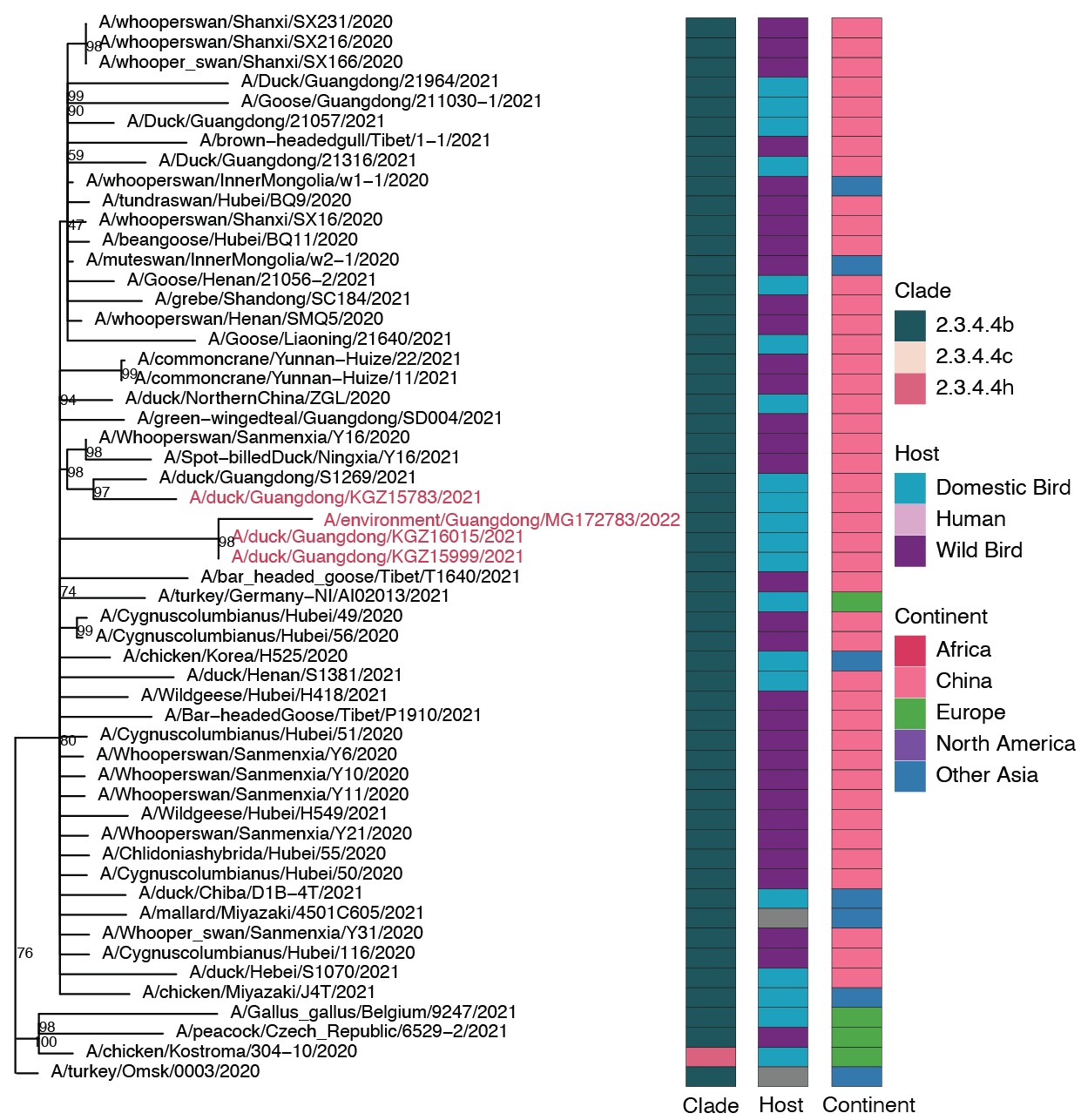
**

**Supplementary Fig. 5. PB2 gene maximum likelihood phylogenetic tree.** Maximum likelihood phylogeny subsampled from all HPAI H5Nx sequences and retaining the ten most HxNx homologous sequences for each poultry market isolate identified via BLAST v2.14.0+ in GISAID (1, 2). The tree was generated by IQ-TREE v2.2.0.7 ultrafast bootstrap with 1000 replicates. Isolates from poultry markets and representative genotype-defining strains are shown with red and yellow tips, respectively. Subtype and host of tips are shown as bars on the right. Relevant bootstrap values used for genotyping analysis are shown in the inset.


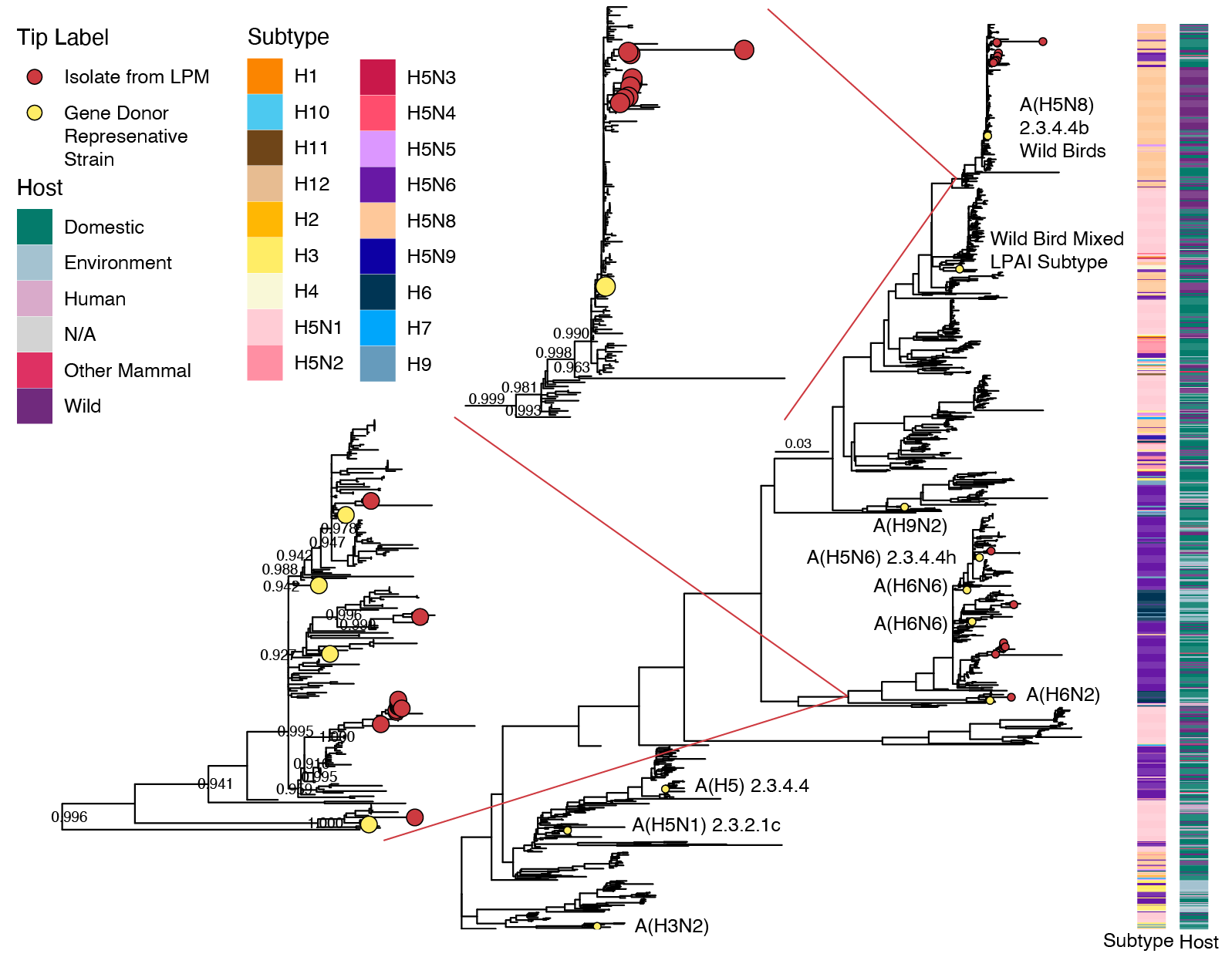


**Supplementary Fig. 6. PB1 gene maximum likelihood phylogenetic tree.** Maximum likelihood phylogeny subsampled from all HPAI H5Nx sequences and retaining the ten most HxNx homologous sequences for each poultry market isolate identified via BLAST v2.14.0+ in GISAID (1, 2). The tree was generated by IQ-TREE v2.2.0.7 ultrafast bootstrap with 1000 replicates. Isolates from poultry markets and representative genotype-defining strains are shown with red and yellow tips, respectively. Subtype and host of tips are shown as bars on the right. Relevant bootstrap values used for genotyping analysis are shown in the inset.


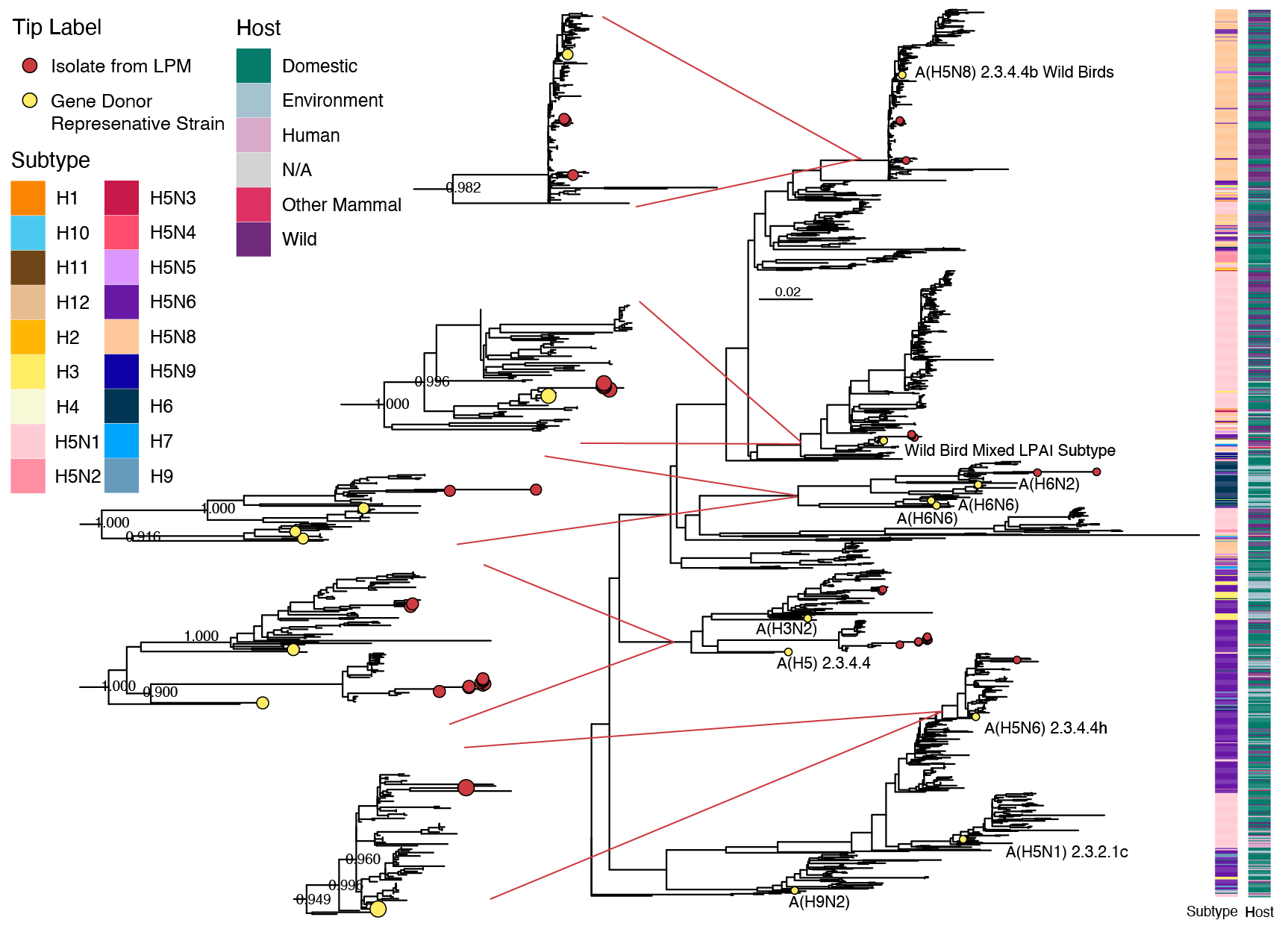


**Supplementary Fig. 7. PA gene maximum likelihood phylogenetic tree.** Maximum likelihood phylogeny subsampled from all HPAI H5Nx sequences and retaining the ten most HxNx homologous sequences for each poultry market isolate identified via BLAST v2.14.0+ in GISAID (1, 2). The tree was generated by IQ-TREE v2.2.0.7 ultrafast bootstrap with 1000 replicates. Isolates from poultry markets and representative genotype-defining strains are shown with red and yellow tips, respectively. Subtype and host of tips are shown as bars on the right. Relevant bootstrap values used for genotyping analysis are shown in the inset.


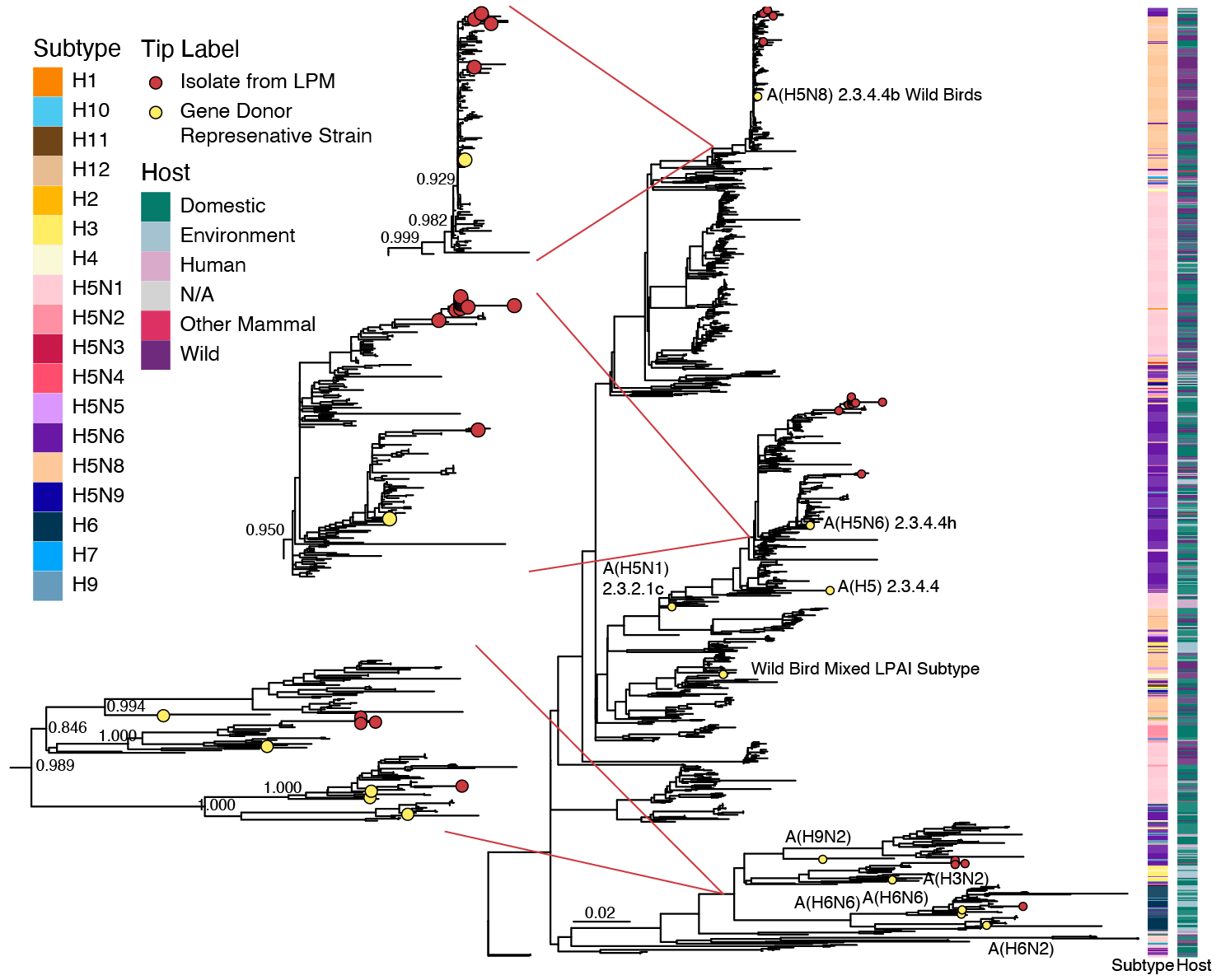


**Supplementary Fig. 8**. **NP gene maximum likelihood phylogenetic tree.** Maximum likelihood phylogeny subsampled from all HPAI H5Nx sequences and retaining the ten most HxNx homologous sequences for each poultry market isolate identified via BLAST v2.14.0+ in GISAID (1, 2). The tree was generated by IQ-TREE v2.2.0.7 ultrafast bootstrap with 1000 replicates. Isolates from poultry markets and representative genotype-defining strains are shown with red and yellow tips, respectively. Subtype and host of tips are shown as bars on the right. Relevant bootstrap values used for genotyping analysis are shown in the inset.


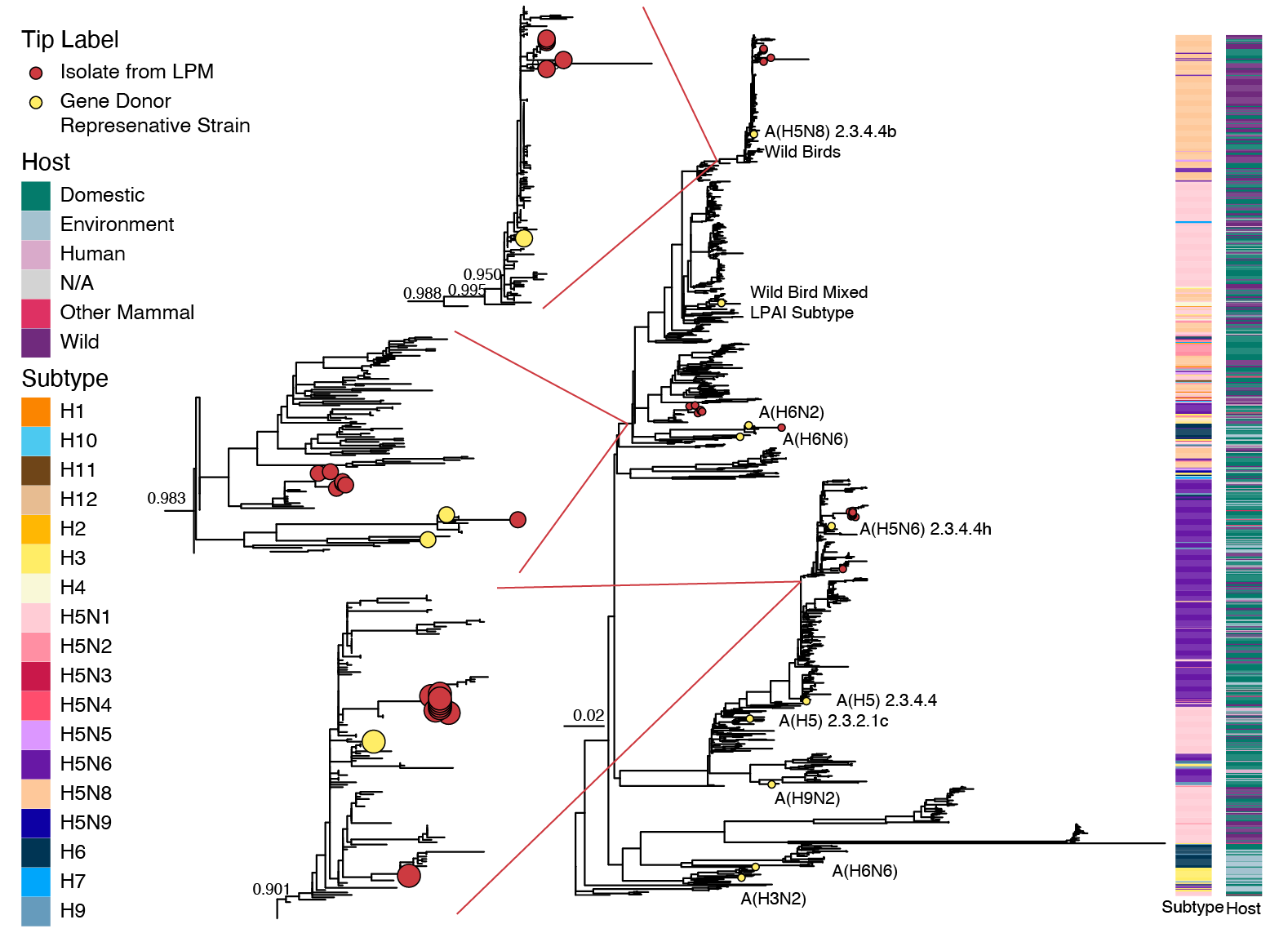


**Supplementary Fig. 9**. **Matrix gene maximum likelihood phylogenetic tree**. Maximum likelihood phylogeny subsampled from all HPAI H5Nx sequences and retaining the ten most HxNx homologous sequences for each poultry market isolate identified via BLAST v2.14.0+ in GISAID (1, 2). The tree was generated by IQ-TREE v2.2.0.7 ultrafast bootstrap with 1000 replicates. Isolates from poultry markets and representative genotype-defining strains are shown with red and yellow tips, respectively. Subtype and host of tips are shown as bars on the right. Relevant bootstrap values used for genotyping analysis are shown in the inset.


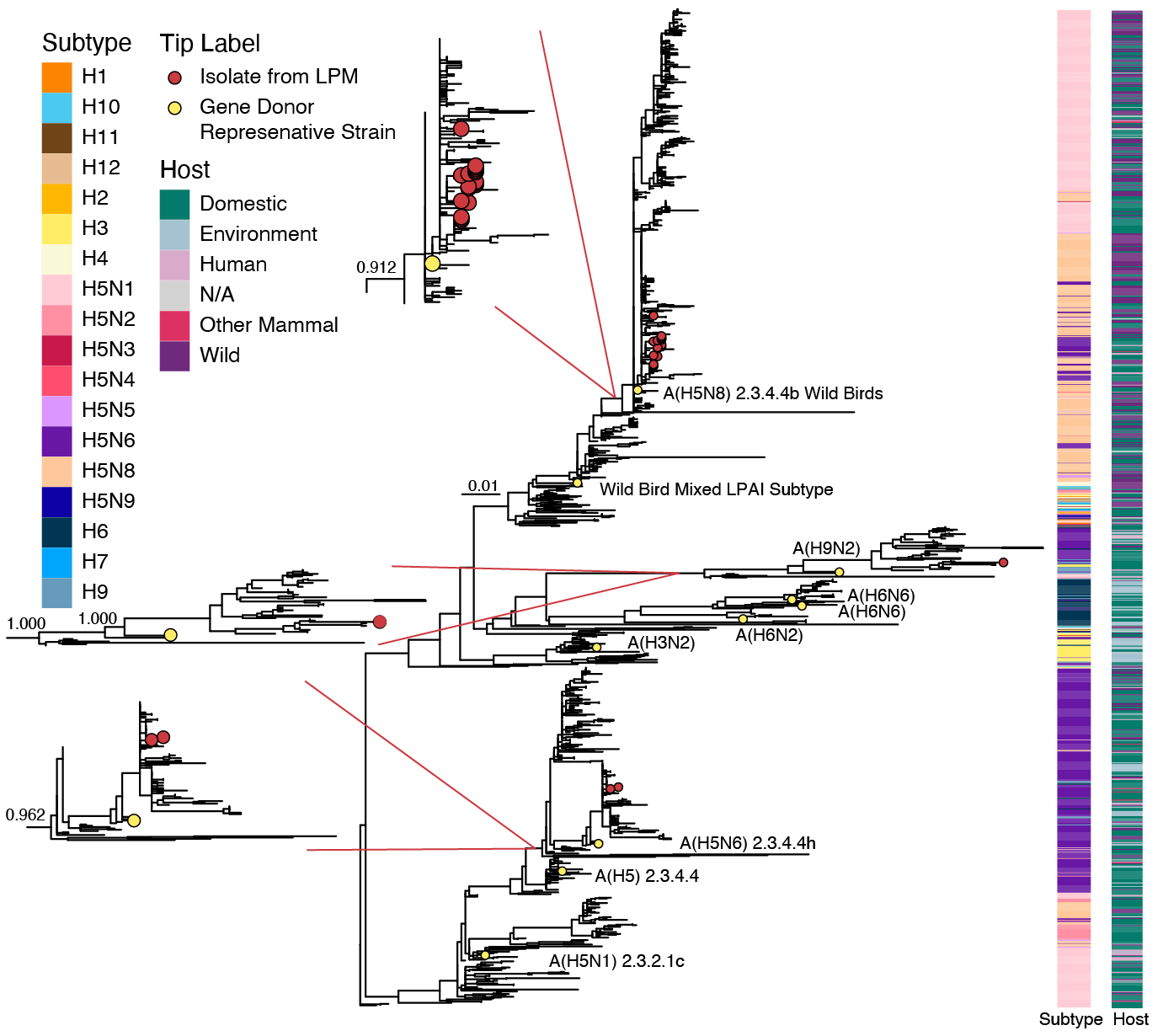


**Supplementary Fig. 10. NS gene maximum likelihood phylogenetic tree** Maximum likelihood phylogeny subsampled from all HPAI H5Nx sequences and retaining the ten most HxNx homologous sequences for each poultry market isolate identified via BLAST v2.14.0+ in GISAID (1, 2). The tree was generated by IQ-TREE v2.2.0.7 ultrafast bootstrap with 1000 replicates. Isolates from poultry markets and representative genotype-defining strains are shown with red and yellow tips, respectively. Subtype and host of tips are shown as bars on the right. Relevant bootstrap values used for genotyping analysis are shown in the inset.


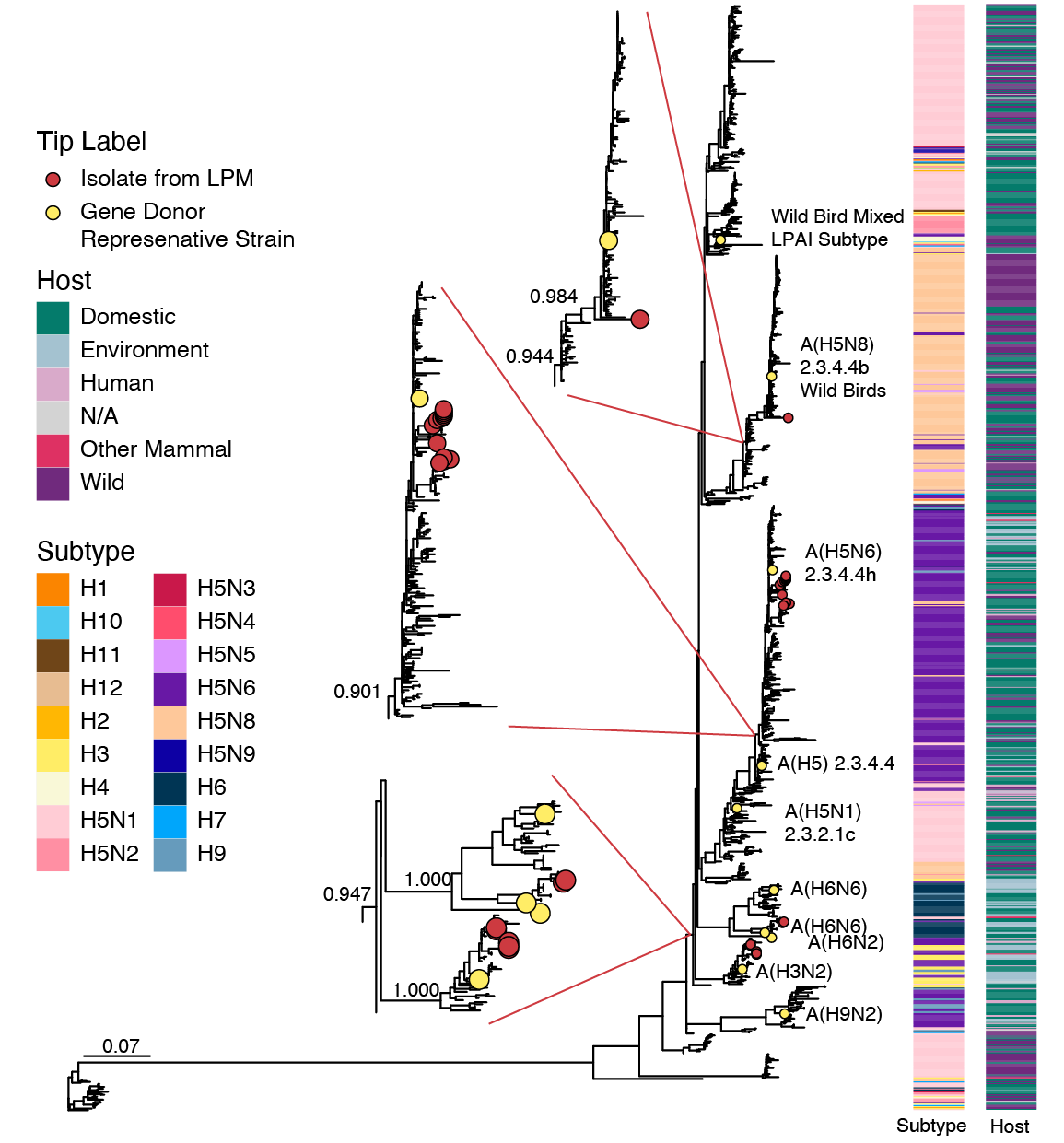


**Supplementary Table 1.** **Molecular markers associated with host adaptation identified in the H5N6 and H5N8 viruses detected in Guangzhou, 2020-2022.** These markers were reported to be association with increased binding to human-type receptors, virulence and transmission in mammals, and antiviral drug resistance (3-7). H3 and N1 numbering were adopted for HA and NA, respectively.

| **Protein** | **Amino acid substitution** | **H5N6 and H5N8 isolates in LPM (N=22)** | **H5N6 (N=18)** | **H5N8 (N=4)** |
| --- | --- | --- | --- | --- |
| HA | PQRERRRKR/GLF | 22 | 18 | 4 |
|  | D101N | 2 | 2 | 0 |
|  | S126N | 0 | 0 | 0 |
|  | S137A | 22 | 18 | 4 |
|  | S138V | 0 | 0 | 0 |
|  | G143R | 0 | 0 | 0 |
|  | S158N | 22 | 18 | 4 |
|  | S159N | 6 | 3 | 3 |
|  | T160A | 22 | 18 | 4 |
|  | N186K/D | 0 | 0 | 0 |
|  | D187G | 0 | 0 | 0 |
|  | E190G | 0 | 0 | 0 |
|  | T192I | 19 | 15 | 4 |
|  | K193R/T | 0 | 0 | 0 |
|  | Q196R | 0 | 0 | 0 |
|  | N197K | 0 | 0 | 0 |
|  | V214I | 0 | 0 | 0 |
|  | G225D | 0 | 0 | 0 |
|  | Q226L | 0 | 0 | 0 |
|  | S227N/R | 21 | 17 | 4 |
|  | G228S | 0 | 0 | 0 |
|  | P239S | 0 | 0 | 0 |
|  | E225K | 0 | 0 | 0 |
|  | K387I | 0 | 0 | 0 |
|  | K393E | 22 | 18 | 4 |
| NA | 55-65 deletion | 13 | 13 | 0 |
|  | V116A | 0 | 0 | 0 |
|  | I117T | 18 | 18 | 0 |
|  | E119A/D/G/V | 0 | 0 | 0 |
|  | Q136L | 0 | 0 | 0 |
|  | R152K | 0 | 0 | 0 |
|  | D198G | 0 | 0 | 0 |
|  | I222M | 0 | 0 | 0 |
|  | S246N | 0 | 0 | 0 |
|  | T247P | 0 | 0 | 0 |
|  | H274Y | 0 | 0 | 0 |
|  | E276D | 0 | 0 | 0 |
|  | E277Q | 0 | 0 | 0 |
|  | R292K | 0 | 0 | 0 |
|  | N294S | 0 | 0 | 0 |
|  | R371K | 0 | 0 | 0 |
|  | S372I | 0 | 0 | 0 |
|  | A401T | 0 | 0 | 0 |
|  | K432T | 0 | 0 | 0 |
| PB2 | D9N | 0 | 0 | 0 |
|  | V25A | 0 | 0 | 0 |
|  | I63T | 0 | 0 | 0 |
|  | M64T | 0 | 0 | 0 |
|  | L89V | 22 | 18 | 4 |
|  | E158G | 0 | 0 | 0 |
|  | E192K | 0 | 0 | 0 |
|  | A199S | 0 | 0 | 0 |
|  | D253N | 0 | 0 | 0 |
|  | D256G | 0 | 0 | 0 |
|  | T271A | 0 | 0 | 0 |
|  | I292V | 18 | 14 | 4 |
|  | G309D | 22 | 18 | 4 |
|  | E358V | 0 | 0 | 0 |
|  | K389R | 22 | 18 | 4 |
|  | L339T | 0 | 0 | 0 |
|  | K482R | 0 | 0 | 0 |
|  | K526R | 0 | 0 | 0 |
|  | M535L | 0 | 0 | 0 |
|  | A588V | 10 | 9 | 1 |
|  | V598T/I | 22 | 18 | 4 |
|  | E627K | 0 | 0 | 0 |
|  | D701N | 0 | 0 | 0 |
|  | S714K | 0 | 0 | 0 |
|  | S715N | 22 | 18 | 4 |
| PB1 | D3V | 22 | 18 | 4 |
|  | N105S | 0 | 0 | 0 |
|  | K207R | 0 | 0 | 0 |
|  | Y436H | 0 | 0 | 0 |
|  | V473L | 0 | 0 | 0 |
|  | K577E | 0 | 0 | 0 |
|  | V598P | 0 | 0 | 0 |
|  | D662G | 22 | 18 | 4 |
|  | T677M | 0 | 0 | 0 |
|  | S678N | 9 | 9 | 0 |
| PB1-F2 | N66S | 6 | 6 | 0 |
| PA | A37S | 0 | 0 | 0 |
|  | I38M/T/S/L | 0 | 0 | 0 |
|  | V63I | 0 | 0 | 0 |
|  | T97I | 0 | 0 | 0 |
|  | K142N/E | 0 | 0 | 0 |
|  | K158R | 0 | 0 | 0 |
|  | P190S | 22 | 18 | 4 |
|  | N356R | 0 | 0 | 0 |
|  | N383D | 22 | 18 | 4 |
|  | Q400P | 22 | 18 | 4 |
|  | N409S | 21 | 17 | 4 |
|  | S421I | 0 | 0 | 0 |
|  | R443K | 0 | 0 | 0 |
|  | K497R | 0 | 0 | 0 |
|  | T515A | 0 | 0 | 0 |
|  | K615N | 0 | 0 | 0 |
| PA-X | Truncation | 0 | 0 | 0 |
| NP | I41V | 0 | 0 | 0 |
|  | Y52N | 0 | 0 | 0 |
|  | K91R | 0 | 0 | 0 |
|  | M105V | 4 | 0 | 4 |
|  | I109T | 0 | 0 | 0 |
|  | A184K | 22 | 18 | 4 |
|  | K198R | 0 | 0 | 0 |
|  | E210D | 0 | 0 | 0 |
|  | K227R | 0 | 0 | 0 |
|  | K229R | 0 | 0 | 0 |
|  | N319K | 0 | 0 | 0 |
|  | E434K | 0 | 0 | 0 |
|  | K470R | 0 | 0 | 0 |
| M1 | N30D | 22 | 18 | 4 |
|  | P41A | 22 | 18 | 4 |
|  | I43M | 22 | 18 | 4 |
|  | T215A | 22 | 18 | 4 |
| M2 | L26F | 0 | 0 | 0 |
|  | V27A | 0 | 0 | 0 |
|  | A30V/T/S | 0 | 0 | 0 |
|  | S31N/G | 1 | 0 | 1 |
|  | G34E | 0 | 0 | 0 |
| NS1 | P42S | 22 | 18 | 4 |
|  | D74N | 0 | 0 | 0 |
|  | 80-84 deletion | 15 | 11 | 4 |
|  | D92E | 13 | 10 | 3 |
|  | L103F | 22 | 18 | 4 |
|  | I106M | 22 | 18 | 4 |
|  | C138F | 17 | 16 | 1 |
|  | V149A | 22 | 18 | 4 |
| NS2/NEP | M161I | 0 | 0 | 0 |

**Supplementary Table 2.** Acknowledgment of influenza A(HxNx) virus gene accession numbers used in phylogenetic trees available on GISAID’s EpiFlu™ Database uploaded up to 30^th^ June 2023.

| **Isolate_Id** | **Isolate_Name** | **Isolate_Id** | **Isolate_Name** | **Isolate_Id** | **Isolate_Name** |
| --- | --- | --- | --- | --- | --- |
| EPI_ISL_10005 | A/chicken/Hebei/326/2005 | EPI_ISL_17768824 | A/Muscovy_duck/Vietnam/HN5046/2018 | EPI_ISL_340795 | A/Env/Guangdong/Huizhou/C18280077/2018-02-22 |
| EPI_ISL_10054 | A/goose/Vietnam/3/05 | EPI_ISL_17768825 | A/Muscovy_duck/Vietnam/HN5047/2018 | EPI_ISL_340797 | A/Env/Guangdong/Huizhou/C18280031/2018-01-22 |
| EPI_ISL_10056 | A/chicken/Guangxi/12/2004 | EPI_ISL_17768826 | A/Muscovy_duck/Vietnam/HN5048/2018 | EPI_ISL_340798 | A/Env/Guangdong/zhongshan/C182870234/2018-04-24 |
| EPI_ISL_10057 | A/duck/Guangxi/13/2004 | EPI_ISL_177761 | A/cat/Sichuan/SC18/2014 | EPI_ISL_340799 | A/Env/Guangdong/Huizhou/C17280804/2017-11-21 |
| EPI_ISL_1009681 | A/chicken/Korea/H541/2020 | EPI_ISL_17777533 | A/peruvian_booby/Peru/LIM-INS-012/2023 | EPI_ISL_340805 | A/Env/Guangdong/Shenzhen/C18061184/2018-05-08 |
| EPI_ISL_1009685 | A/duck/Korea/H549/2020 | EPI_ISL_17785726 | A/swan/Austria/22004012-001/2022 | EPI_ISL_340806 | A/Env/Guangdong/Qingyuan/C18285099/2018-02-28 |
| EPI_ISL_1009706 | A/wild_bird/Korea/H379/2020 | EPI_ISL_17785730 | A/mute_swan/Austria/21155360-001/2021 | EPI_ISL_340807 | A/Env/Guangdong/Foshan/C182750200/2018-03-06 |
| EPI_ISL_1009710 | A/wild_duck/Korea/H331/2020 | EPI_ISL_177869 | A/chicken/TongHai/302/2014 | EPI_ISL_340808 | A/Env/Guangdong/Foshan/C182753007/2018-01-02 |
| EPI_ISL_10107 | A/turkey/Turkey/1/2005 | EPI_ISL_17791548 | A/domestic_duck/England/069592/2023 | EPI_ISL_340809 | A/Env/Guangdong/Foshan/C172750608/2017-12-26 |
| EPI_ISL_10228 | A/chicken/Vietnam/398/2005 | EPI_ISL_17791550 | A/domestic_duck/England/069603/2023 | EPI_ISL_340810 | A/Env/Guangdong/Foshan/C172750600/2017-12-26 |
| EPI_ISL_1038924 | A/Astrakhan/3212/2020 | EPI_ISL_17791553 | A/chicken/England/062649/2023 | EPI_ISL_340811 | A/Env/Guangdong/Foshan/C172750528/2017-12-05 |
| EPI_ISL_1041150 | A/duck/Chiba/D1B-4T/2021 | EPI_ISL_17805252 | A/chicken/Guangdong/684/2022 | EPI_ISL_340816 | A/Env/Guangdong/zhongshan/C182870028/2018-01-15 |
| EPI_ISL_10413 | A/duck/Laos/3295/2006 | EPI_ISL_17805259 | A/chicken/Guangdong/567/2022 | EPI_ISL_340823 | A/Env/Guangdong/zhanjiang/C18277136/2018-04-02 |
| EPI_ISL_10576444 | A/Anser_anser/Belgium/1809_0002/2022 | EPI_ISL_17805291 | A/chicken/Guangdong/310/2022 | EPI_ISL_340824 | A/Env/Guangdong/Foshan/C182750085/2018-01-30 |
| EPI_ISL_1063533 | A/Mandarin_duck/Kagoshima/KU-d57/2020 | EPI_ISL_17805905 | A/duck/Japan/KU-d89/2021 | EPI_ISL_340830 | A/Env/Guangdong/zhanjiang/C17277335/2017-11-27 |
| EPI_ISL_10650 | A/Japanese_white-eye/Hong_Kong/1038/2006 | EPI_ISL_17809478 | A/duck/Vietnam/HN6893/2021 | EPI_ISL_340831 | A/Env/Guangdong/Shaoguan/C17272470/2018-01-16 |
| EPI_ISL_10666 | A/duck/Guiyang/2231/2005 | EPI_ISL_17811768 | A/Eastern_Spot-billed_Duck/Shanghai/JDS19510/2019 | EPI_ISL_340832 | A/Env/Guangdong/zhanjiang/C18277135/2018-04-02 |
| EPI_ISL_10667 | A/goose/Guiyang/337/2006 | EPI_ISL_17811769 | A/Falcated_Duck/Shanghai/JDS19603/2019 | EPI_ISL_340833 | A/Env/Guangdong/Foshan/C182750020/2018-01-02 |
| EPI_ISL_10668 | A/chicken/Guiyang/441/2006 | EPI_ISL_17811770 | A/Common_Teal/Shanghai/JDS19625/2019 | EPI_ISL_340838 | A/Env/Guangdong/C17059521/YF/2017-11-14 |
| EPI_ISL_10706 | A/goose/Shantou/3265/2006 | EPI_ISL_17811771 | A/Eastern_Spot-billed_Duck/Shanghai/JDS19701/2019 | EPI_ISL_340844 | A/Env/Guangdong/C17285752/QY/2017-11-21 |
| EPI_ISL_10737 | A/goose/Guangxi/3017/2005 | EPI_ISL_178261 | A/Yunnan/14563/2015 | EPI_ISL_340846 | A/Env/Guangdong/zhongshan/C172870590/2017-11-28 |
| EPI_ISL_10753 | A/duck/Yunnan/4400/2005 | EPI_ISL_17851876 | A/chicken/Ohio/OH22-26275-1/2022 | EPI_ISL_340847 | A/Duck/Guangdong/Yangjiang/PO18284009/2018-01-09 |
| EPI_ISL_10765 | A/chicken/Guiyang/3055/2005 | EPI_ISL_17851883 | A/chicken/Japan/AZRC-TU-17/2022 | EPI_ISL_340848 | A/Env/Guangdong/C172790591/ZHQ/2017-5-8 |
| EPI_ISL_10766 | A/duck/Guiyang/3242/2005 | EPI_ISL_17851901 | A/chicken/Ohio/OH22-7075/2022 | EPI_ISL_340852 | A/Goose/Guangdong/PO1707260055/GZH/2017 |
| EPI_ISL_10768 | A/chicken/Guiyang/3570/2005 | EPI_ISL_17885921 | A/pelican/Maule/231155-2/2023 | EPI_ISL_340853 | A/Env/Guangdong/C17272335/SHG/2017-5-16 |
| EPI_ISL_1081369 | A/Chongqing/00013/2021 | EPI_ISL_17885991 | A/black_vulture/Georgia/W22-406/2022 | EPI_ISL_344546 | A/Duck/Egypt/AR560/2018 |
| EPI_ISL_1081370 | A/Anhui/2021-00011/2020 | EPI_ISL_179175 | A/muscovy_duck/Quang_Ninh/4c111/2013 | EPI_ISL_351792 | A/Chicken/Egypt/Al00994/2019 |
| EPI_ISL_11007530 | A/swan/Slovenia/2049_22VIR777-3/2021 | EPI_ISL_179634 | A/duck/Jiangxi/NCDZT1123/2014 | EPI_ISL_354560 | A/Chicken/Jiangxi/45153/2016 |
| EPI_ISL_1114735 | A/chicken/Tyumen/302-01/2020 | EPI_ISL_179647 | A/duck/Sichuan/NCXJ15/2014 | EPI_ISL_354562 | A/Chicken/Jiangxi/45093/2016 |
| EPI_ISL_1114745 | A/chicken/Kostroma/304-10/2020 | EPI_ISL_17964849 | A/great_horned_owl/Colorado/23-004706-001-original/2023 | EPI_ISL_354571 | A/Environment/Jiangxi/24995/2014 |
| EPI_ISL_11208193 | A/duck/Guangxi/S30428/2021 | EPI_ISL_17964851 | A/great_horned_owl/Montana/23-004958-001-original/2023 | EPI_ISL_355938 | A/green_sandpiper/Kurgan/1050/2018 |
| EPI_ISL_11208194 | A/duck/Guangxi/S31116/2021 | EPI_ISL_17964878 | A/red-tailed_hawk/California/23-006644-003-original/2023 | EPI_ISL_363823 | A/wild_bird/Hunan/01.14_YYDTHF2-25-2/2016 |
| EPI_ISL_11208195 | A/duck/Guizhou/S4702/2021 | EPI_ISL_17964888 | A/crow/Minnesota/23-007350-001-original/2023 | EPI_ISL_366237 | A/Cygnus_olor/England/WVUK/2016 |
| EPI_ISL_11208196 | A/duck/Hunan/S40199/2021 | EPI_ISL_17964890 | A/American_crow/Iowa/23-007468-001-original/2023 | EPI_ISL_366635 | A/chicken/Alkharj/910/2018 |
| EPI_ISL_11208197 | A/duck/Hunan/S40268/2021 | EPI_ISL_17964894 | A/Canada_goose/Washington/23-007799-001-original/2023 | EPI_ISL_368668 | A/duck/Viet_Nam/HU1-2361/2014 |
| EPI_ISL_11208198 | A/duck/Yunnan/S4318/2021 | EPI_ISL_17964921 | A/glaucous_gull/California/23-010132-001-original/2023 | EPI_ISL_369325 | A/mallard/Korea/A32-3/2017 |
| EPI_ISL_11208199 | A/duck/Zhejiang/S4854/2021 | EPI_ISL_17964939 | A/great_horned_owl/Montana/23-011850-001-original/2023 | EPI_ISL_369326 | A/mallard/Korea/A42-4/2017 |
| EPI_ISL_11208200 | A/goose/Guangdong/S4751/2021 | EPI_ISL_17964941 | A/sanderling/Oregon/23-011964-001-original/2023 | EPI_ISL_369327 | A/spot-billed_duck/Korea/A45-1/2017 |
| EPI_ISL_11211731 | A/duck/Bangladesh/19D1818/2021 | EPI_ISL_17973458 | A/wildbird/Ecuador/IC06-4590/2023 | EPI_ISL_369328 | A/mallard/Korea/A46-1-4/2017 |
| EPI_ISL_1122425 | A/chicken/England/043315/2020 | EPI_ISL_17982387 | A/Common_Tern/Netherlands/10/2023 | EPI_ISL_372812 | A/goose/China/GS42-1.seq/2016 |
| EPI_ISL_1123358 | A/brent_goose/England/095684/2020 | EPI_ISL_18000337 | A/Jiangxi/16791/2022 | EPI_ISL_3770721 | A/GX-hechi/01/2021 |
| EPI_ISL_1139015 | A/wild_bird/Netherlands/20016515-002/2020 | EPI_ISL_18007229 | A/large-billed_crow/Hokkaido/B052/2023 | EPI_ISL_3770740 | A/GX-guilin/11151/2021 |
| EPI_ISL_11392591 | A/peregrine_falcon/Northern_Ireland/AI102021-2/2021 | EPI_ISL_18011225 | A/black-headed_gull/Finland/7826_23VIR6803-11/2023 | EPI_ISL_378253 | A/duck/Nha_Trang/194/2017 |
| EPI_ISL_11402318 | A/turkey/England/018179/2021 | EPI_ISL_18011232 | A/white-tailed_eagle/Finland/7800_23VIR6803-10/2023 | EPI_ISL_381810 | A/Egyptian_goose/South_Africa/001/2017 |
| EPI_ISL_11408 | A/chicken/India/NIV33487/06 | EPI_ISL_18012465 | A/swan/Hebei/B01/2021 | EPI_ISL_3849 | A/Ck/HK/YU777/02 |
| EPI_ISL_11449674 | A/Gallus_gallus/Belgium/3194_0001/2022 | EPI_ISL_18033192 | A/mute_swan/Poland/MB113/2023 | EPI_ISL_386923 | A/Streptopelia_decaocto/Jiangxi/E1/2015 |
| EPI_ISL_11504587 | A/chicken/Vietnam/HU14-LB11/2021 | EPI_ISL_18033195 | A/laying_hen/Poland/H126-KL2/2023 | EPI_ISL_386925 | A/quail/Jiangxi/B9/2015_H5N6 |
| EPI_ISL_11560325 | A/Great_black-backed_Gull/Netherlands/3/2022 | EPI_ISL_18033205 | A/black-headed_gull/Poland/MB141-T/2023 | EPI_ISL_386927 | A/Mallard/Jiangxi/JXH9/2014_H5N6 |
| EPI_ISL_11561589 | A/chicken/England/011981/2022 | EPI_ISL_18033208 | A/black-headed_gull/Poland/MB145/2023 | EPI_ISL_386929 | A/chickenl/Jiangxi/E84/2014 |
| EPI_ISL_11561592 | A/turkey/England/016515/2022 | EPI_ISL_18033233 | A/black-headed_gull/Poland/MB210/2023 | EPI_ISL_387351 | A/duck/Viet_Nam/HN-2431/2015 |
| EPI_ISL_11633613 | A/Cygnus_columbianus/Hubei/117/2021 | EPI_ISL_18033422 | A/chicken/Poland/H296/2023 | EPI_ISL_389022 | A/chicken/Nghe_An/01VTC/2018 |
| EPI_ISL_11871 | A/Egypt/2321-NAMRU3/2007 | EPI_ISL_18058053 | A/duck/Shandong/SD0261/2021 | EPI_ISL_389082 | A/chicken/Nghe_An/14VTC/2015 |
| EPI_ISL_11922808 | A/duck/Poland/H188_22VIR2515-2/2022 | EPI_ISL_18058054 | A/duck/Shandong/SD0263/2021 | EPI_ISL_389738 | A/chicken/Egypt/H13791C/2017 |
| EPI_ISL_11922818 | A/swan/Romania/10455_22VIR2749-4/2022 | EPI_ISL_18058055 | A/duck/Sichuan/SC4822/2021 | EPI_ISL_389756 | A/chicken/Egypt/Q13941B/2017 |
| EPI_ISL_11922819 | A/laying_hen/Romania/10470_22VIR2749-5/2022 | EPI_ISL_18058056 | A/duck/Sichuan/SC5698/2021 | EPI_ISL_3907 | A/duck/Guangxi/50/2001 |
| EPI_ISL_11971490 | A/pheasant/New_York/22-008760-008/2022 | EPI_ISL_18058692 | A/black-headed_gull/Austria/23072665-007/2023 | EPI_ISL_390772 | A/EN/Hunan/11043/2016 |
| EPI_ISL_11971491 | A/mallard/New_York/22-008760-007/2022 | EPI_ISL_18065335 | A/chicken/England/089930/2023 | EPI_ISL_390787 | A/EN/Hunan/03259/2015 |
| EPI_ISL_121541 | A/Indonesia/5/2005 | EPI_ISL_18065338 | A/kittiwake/Scotland/091080/2023 | EPI_ISL_3909 | A/duck/Zhejiang/52/2000 |
| EPI_ISL_12174842 | A/Ezo_red_fox/Hokkaido/1/2022 | EPI_ISL_18065346 | A/common_gull/England/127517/2023 | EPI_ISL_393488 | A/duck/Moscow/5586/2018 |
| EPI_ISL_12325210 | A/chicken/Czech_Republic/3306-2/2022 | EPI_ISL_18065393 | A/common_tern/England/086637/2023 | EPI_ISL_399464 | A/Anas_platyrhynchos/Belgium/9594H191810/2016 |
| EPI_ISL_12471661 | A/pelican/Greece/64_SP_22VIR3126-7/2022 | EPI_ISL_180754 | A/environment/Hubei/950/2013 | EPI_ISL_399630 | A/chicken/Egypt/V1410/2018 |
| EPI_ISL_1254 | A/Goose/Guangdong/1/96 | EPI_ISL_18075722 | A/mediterranean_gull/Spain/2886-14-2023_23VIR6502-31/2023 | EPI_ISL_400266 | A/Common_Teal/Amur_region/31b/2019 |
| EPI_ISL_12572652 | A/chicken/Anhui/S1740/2022 | EPI_ISL_18076012 | A/pheasant/Italy/23VIR6483-3/2023 | EPI_ISL_400274 | A/mallard/Novosibirsk_region/1894k/2019 |
| EPI_ISL_12572654 | A/duck/Guangdong/S4518/2021 | EPI_ISL_18090351 | A/canine/China/GX30/2023 | EPI_ISL_400485 | A/Green_pheasant/Hunan/10/2015 |
| EPI_ISL_12572655 | A/duck/Guangdong/S4525/2021 | EPI_ISL_18094395 | A/black-headed_gull/Leningrad_region/RII-WD263M/2023 | EPI_ISL_400486 | A/Common_pheasant/Hunan/11/2015 |
| EPI_ISL_12572656 | A/duck/Guizhou/S1321/2022 | EPI_ISL_18102700 | A/feline/South_Korea/SNU-01/2023 | EPI_ISL_400492 | A/Greylag_goose/Hunan/1/2017 |
| EPI_ISL_12572659 | A/duck/Hubei/SE220/2022 | EPI_ISL_18102701 | A/feline/South_Korea/SNU-02/2023 | EPI_ISL_4031415 | A/Duck/China/B2_NS/2018 |
| EPI_ISL_12572661 | A/goose/Guizhou/S1541/2022 | EPI_ISL_181094 | A/eurasian_wigeon/Netherlands/2/2014 | EPI_ISL_4031416 | A/Duck/China/B3_NS/2019 |
| EPI_ISL_12572662 | A/goose/Hunan/SE284/2022 | EPI_ISL_18112327 | A/black-headed_gull/Wales/094394/2023 | EPI_ISL_4031419 | A/Duck/China/B6_NS/2019 |
| EPI_ISL_12572663 | A/pigeon/Jiangxi/S40784/2021 | EPI_ISL_18112331 | A/chicken/Jersey/095598/2023 | EPI_ISL_4031428 | A/Duck/China/B6_PA/2019 |
| EPI_ISL_12690589 | A/chicken/Iowa/22-009287-001/2022 | EPI_ISL_18112334 | A/black-headed_gull/England/354816/2023 | EPI_ISL_4031437 | A/Duck/China/B6_PB1/2019 |
| EPI_ISL_12691035 | A/chicken/North_Dakota/22-009337-002/2022 | EPI_ISL_18118722 | A/Black-headed_Gull/Netherlands/115/2023 | EPI_ISL_4031446 | A/Duck/China/B6_PB2/2019 |
| EPI_ISL_1290892 | A/duck/Laos/2310/2019 | EPI_ISL_18122416 | A/human/YunnanKM/352/2019 | EPI_ISL_4031450 | A/Chicken/Viet_Nam/AI-1606/2016 |
| EPI_ISL_13048382 | A/avian/Burkina_Faso/21VIR11911-3/2021 | EPI_ISL_18132358 | A/Owl/Texas/USDA-012996-001/2022 | EPI_ISL_4032203 | A/chicken/Changhua/15120008/2015 |
| EPI_ISL_13048383 | A/chicken/Burkina_Faso/21VIR11911-5/2021 | EPI_ISL_18132985 | A/Backyard_bird/Iowa/USDA-012510-003/2022 | EPI_ISL_4032206 | A/chicken/Changhua/17030063-1/2017 |
| EPI_ISL_13243350 | A/duck/Bangladesh/19D1874/2022 | EPI_ISL_18133080 | A/Backyard_bird/Maine/USDA-007582-001/2022 | EPI_ISL_4032218 | A/chicken/Chiayi/18120003-1/2018 |
| EPI_ISL_13243697 | A/environment/England/030642/2020 | EPI_ISL_18133459 | A/goose/New_Hampshire/USDA-010920-002/2022 | EPI_ISL_4032219 | A/chicken/Hsinchu/16080004-2/2016 |
| EPI_ISL_13295137 | A/duck/Cambodia/c18MKAP189/2018 | EPI_ISL_18137626 | A/wildbird/Ecuador/7607/2023 | EPI_ISL_4032220 | A/chicken/Kaohsiung/16010047/2016 |
| EPI_ISL_13295138 | A/duck/Cambodia/e10T241C18/2020 | EPI_ISL_18161267 | A/european_herring_gull/Leningrad_region/RII-WD392S/2023 | EPI_ISL_4032222 | A/chicken/Kaohsiung/16040021-1/2016 |
| EPI_ISL_13352712 | A/mandarin_duck/Heilongjiang/HL-1/2021 | EPI_ISL_182069 | A/environment/Jiangxi/10164/2014 | EPI_ISL_4032224 | A/chicken/Kaohsiung/16120012/2016 |
| EPI_ISL_13352726 | A/whooper_swan/Henan/14/2021 | EPI_ISL_18219237 | A/Anser_anser/France/22P007390/2022 | EPI_ISL_4032235 | A/chicken/Kaohsiung/18080004-1/2018 |
| EPI_ISL_13369742 | A/mute_swan/England/298902/2021 | EPI_ISL_1822592 | A/whooper_swan/Shanxi/SX166/2020 | EPI_ISL_4032238 | A/chicken/Miaoli/18040011-1/2018 |
| EPI_ISL_13370511 | A/turkey/Wales/065047/2021 | EPI_ISL_1822593 | A/whooper_swan/Shanxi/SX206/2020 | EPI_ISL_4032276 | A/chicken/Tainan/15020214/2015 |
| EPI_ISL_13370571 | A/chicken/England/069816/2021 | EPI_ISL_1822594 | A/whooper_swan/Shanxi/SX216/2020 | EPI_ISL_4032279 | A/chicken/Tainan/18040014-2/2018 |
| EPI_ISL_13418526 | A/Little_egret/Israel/172/2022 | EPI_ISL_1822595 | A/whooper_swan/Shanxi/SX231/2020 | EPI_ISL_4032284 | A/chicken/Taipei_City/18050010-8/2018 |
| EPI_ISL_13432603 | A/turkey/South_Dakota/22-010139-001/2022 | EPI_ISL_18244181 | A/mute_swan/Poland/MB021-21-22VIR5675-11/2021 | EPI_ISL_4032296 | A/chicken/Yunlin/15050012/2015 |
| EPI_ISL_13434407 | A/chicken/Montana/22-010445-001/2022 | EPI_ISL_18245790 | A/swan/Poland/MB058-22_22VIR5675-2/2022 | EPI_ISL_4032304 | A/chicken/Yunlin/16050053/2016 |
| EPI_ISL_13453568 | A/turkey/England/081490/2022 | EPI_ISL_1829205 | A/whooper_swan/Henan/SMQ5/2020 | EPI_ISL_4032313 | A/chicken/Yunlin/17030014-1/2017 |
| EPI_ISL_13465456 | A/Great_white_pelican/Israel/619/2021 | EPI_ISL_1834185 | A/whooper_swan/Henan/SMQ6/2020 | EPI_ISL_4032324 | A/chicken/Yunlin/17060019/2017 |
| EPI_ISL_13519451 | A/gull/France/22P015977/2022 | EPI_ISL_190641 | A/chicken/Vietnam/NCVD-03/2008 | EPI_ISL_4032330 | A/chicken/Yunlin/18020003-2/2018 |
| EPI_ISL_135216 | A/Guizhou/1/2013 | EPI_ISL_191765 | A/scarlet_ibis/Germany/AR44-L01279/2015 | EPI_ISL_4032434 | A/duck/Pingtung/17A00408-1-10T/2017 |
| EPI_ISL_136169 | A/duck/Vietnam/NCVD-1584/2012 | EPI_ISL_1937884 | A/whooper_swan/Henan/SM1/2020 | EPI_ISL_4032531 | A/duck/Yunlin/15010538/2015 |
| EPI_ISL_137553 | A/chicken/Hong_Kong/AP156/2008 | EPI_ISL_1938307 | A/whooper_swan/Henan/SM86/2020 | EPI_ISL_4032534 | A/duck/Yunlin/15A04883-1-10T/2015 |
| EPI_ISL_1379443 | A/chicken/Vietnam/Raho4-Cd-20-421/2020 | EPI_ISL_1939617 | A/eurasian_eagle-owl/Henan/SMQ11/2020 | EPI_ISL_4032535 | A/duck/Yunlin/15A5019/2015 |
| EPI_ISL_139386 | A/quail/Jiangsu/k0104/2010 | EPI_ISL_1941444 | A/duck/Czech_Republic/7681-7/2021 | EPI_ISL_4032536 | A/duck/Yunlin/17110001/2017 |
| EPI_ISL_13955171 | A/chicken/Czech_Republic/2968/2022 | EPI_ISL_195308 | A/chicken/Yunnan/19/2015 | EPI_ISL_4032540 | A/duck/Yunlin/18060006-1/2018 |
| EPI_ISL_13957819 | A/chicken/Mali/T1-177_22VIR6104-1/2022 | EPI_ISL_198754 | A/duck/Hunan/12.17_YYFQH012-O/2014 | EPI_ISL_4032569 | A/goose/Tainan/17030024-1/2017 |
| EPI_ISL_13969430 | A/Mute_swan/Wales/058560/2022 | EPI_ISL_198757 | A/goose/Jilin/04.04_SY003-O/2015 | EPI_ISL_4032573 | A/goose/Taoyuan/18120002-2/2018 |
| EPI_ISL_139735 | A/chicken/Indonesia/BL/2003 | EPI_ISL_198814 | A/duck/Hunan/02.26_YYFQH297-P/2014 | EPI_ISL_404988 | A/chicken/Thanh_Hoa/13836VTC/2019 |
| EPI_ISL_13990714 | A/wild_bird/Spain/1302-1_22VIR6312-11/2022 | EPI_ISL_198828 | A/duck/Hunan/01.16_YYFQH315-P/2014 | EPI_ISL_404993 | A/white-fronted_goose/Germany-BB/AI00018/2020 |
| EPI_ISL_13990735 | A/turkey/Spain/1097-7_22VIR6312-36/2022 | EPI_ISL_198833 | A/duck/Hunan/12.17_YYGK014/2014 | EPI_ISL_405391 | A/chicken/Czech_Republic/1175-1/2020 |
| EPI_ISL_14171729 | A/Eurasian_Spoonbill/Netherlands/1A/2022 | EPI_ISL_198857 | A/duck/Hunan/12.17_YYGK040/2014 | EPI_ISL_4055128 | A/goose/Yunlin/18090006-1/2018 |
| EPI_ISL_14171741 | A/Eurasian_Spoonbill/Netherlands/1B/2022 | EPI_ISL_198863 | A/duck/Hunan/12.17_YYGK0057/2014 | EPI_ISL_4061478 | A/duck/Vietnam/1914/2014 |
| EPI_ISL_14174306 | A/goose/China/0701/2018 | EPI_ISL_198873 | A/duck/Hunan/12.17_YYGK079-1/2014 | EPI_ISL_4061712 | A/whooper_swan/Miyagi/0402B001/2021 |
| EPI_ISL_14174523 | A/swine/China/RZ/2018 | EPI_ISL_198884 | A/chicken/Jiangxi/12.05_NCDZT0012/2014 | EPI_ISL_4061714 | A/white-tailed_eagle/Hokkaido/20210127001/2021 |
| EPI_ISL_14233919 | A/Eurasian_Spoonbill/Netherlands/3/2022 | EPI_ISL_198888 | A/chicken/Jiangxi/12.05_NCDZT0032/2014 | EPI_ISL_4062440 | A/goose/China/21FU004/2020 |
| EPI_ISL_143542 | A/Vietnam/HN36282/2010 | EPI_ISL_198893 | A/pigeon/Sichuan/04.27_NBXJ743/2014 | EPI_ISL_4062442 | A/goose/China/21FU006/2020 |
| EPI_ISL_14389524 | A/Anser_anser_domesticus/Belgium/1668_0016/2022 | EPI_ISL_198953 | A/environment/Jiangxi/05.07_NCJD0010D/2015 | EPI_ISL_4062444 | A/goose/China/21FU008/2020 |
| EPI_ISL_14391865 | A/Buteo_buteo/Belgium/2606_0006/2022 | EPI_ISL_198985 | A/chicken/Jiangxi/05.06_NCDZT0058B-P/2015 | EPI_ISL_4069650 | A/chicken/Egypt/N16732/2019 |
| EPI_ISL_14393465 | A/Branta_canadensis/Belgium/4821_0001/2022 | EPI_ISL_199011 | A/chicken/Hubei/03.06__WHWTZ0115-O/2015 | EPI_ISL_4070019 | A/egret/Zhejiang/W15/2017 |
| EPI_ISL_14497837 | A/domestic_duck/England/100990/2022 | EPI_ISL_199037 | A/duck/Yunnan/03.16_DQXYL0013-O/2015 | EPI_ISL_4070022 | A/common_buzzard/Denmark/4079-1p1/2017 |
| EPI_ISL_14760611 | A/goose/Italy/IZSLT_21VIR10273/2021 | EPI_ISL_199053 | A/duck/Hunan/01.21_YYFQH015-O/2015 | EPI_ISL_4071001 | A/duck/South_Korea/D14-19/2014 |
| EPI_ISL_14760616 | A/chicken/Italy/21VIR10000/2021 | EPI_ISL_199055 | A/duck/Hunan/01.21_YYFQH018-O/2015 | EPI_ISL_4071213 | A/Mallard__Anas_platyrhynchos_/South_Korea/KNU2019-33/2019 |
| EPI_ISL_14760849 | A/turkey/Italy/21VIR11509/2021 | EPI_ISL_199056 | A/duck/Hunan/01.21_YYFQH019-P/2015 | EPI_ISL_4071308 | A/duck/Mongolia/MS2018-6/2018 |
| EPI_ISL_14761318 | A/laying_hen/Italy/22VIR204-1/2022 | EPI_ISL_199062 | A/Phalacrocorax/Hubei/01.09_V5/2015 | EPI_ISL_4071932 | A/Muscovy_duck/China/H5N6/2020 |
| EPI_ISL_14761327 | A/broiler/Italy/22VIR278-3/2022 | EPI_ISL_199063 | A/Phalacrocorax/Hubei/01.09_V7/2015 | EPI_ISL_4072004 | A/duck/Mongolia/MN18-1/2018 |
| EPI_ISL_14770020 | A/chicken/Egypt/Q16684C/2019 | EPI_ISL_199067 | A/duck/Hunan/02.07_YYFQH026-O/2015 | EPI_ISL_4072162 | A/Eurasian_teal/South_Korea/JB32-15/2019 |
| EPI_ISL_14770172 | A/wild_duck/Shandong/W3580/2020 | EPI_ISL_199071 | A/duck/Hunan/01.21_YYFQH032-P/2015 | EPI_ISL_4073173 | A/duck/Bangladesh/44484/2020 |
| EPI_ISL_14778363 | A/Wild_bird/China/Cixi02/2020 | EPI_ISL_199072 | A/duck/Hunan/02.07_YYFQH032-O/2015 | EPI_ISL_408877 | A/duck/Korea/H2388/2015 |
| EPI_ISL_14819895 | A/Mallard/Ningxia/BY246/2020 | EPI_ISL_199085 | A/chicken/Yunnan/03.15_DQJT0054-Z-P/2015 | EPI_ISL_408920 | A/chicken/Korea/H2514/2015 |
| EPI_ISL_14819899 | A/Wild_Duck/Ningxia/Y99/2021 | EPI_ISL_199102 | A/chicken/Yunnan/03.16__DQXYL0050-O/2015 | EPI_ISL_412997 | A/chicken/Iran/18VIR2027-04/2017 |
| EPI_ISL_14819900 | A/Spot-billed_Duck/Ningxia/Y16/2021 | EPI_ISL_199104 | A/chicken/Yunnan/03.16_DQXYL0052-O/2015 | EPI_ISL_413008 | A/flamingo/Iran/17RS654-18/2016 |
| EPI_ISL_14822356 | A/Spot-billed_Duck/Ningxia/Y26/2021 | EPI_ISL_199120 | A/duck/Hunan/04.14_YYGK464-O/2015 | EPI_ISL_413010 | A/little_grebe/Iran/17RS654-10/2016 |
| EPI_ISL_14822357 | A/Wild_Duck/Ningxia/Y54/2021 | EPI_ISL_199156 | A/goose/Shandong/12.08_YG-GS/2014 | EPI_ISL_413226 | A/Mallard/Republic_of_Georgia/2/2015 |
| EPI_ISL_14822426 | A/Bar-headed_Goose/Tibet/P2320/2021 | EPI_ISL_199160 | A/goose/Shandong/01.01_JY-GS/2015 | EPI_ISL_4177 | A/Ck/HK/WF157/2003 |
| EPI_ISL_14822452 | A/Bar-headed_Goose/Tibet/P1910/2021 | EPI_ISL_199174 | A/duck/Guangdong/03.26_DGCPLB021-O/2015 | EPI_ISL_4186 | A/Dk/HN/303/2004 |
| EPI_ISL_14822549 | A/bar_headed_goose/Tibet/T1640/2021 | EPI_ISL_199179 | A/duck/Guangdong/03.26_DGCP079-O/2015 | EPI_ISL_419212 | A/chicken/Bulgaria/Dobrich/12-1/2018 |
| EPI_ISL_14823405 | A/Wild_geese/Hubei/H358/2021 | EPI_ISL_199215 | A/duck/Guangdong/04.22_DGCP069-O/2015 | EPI_ISL_419348 | A/chicken/Bulgaria/Haskovo/411/2017 |
| EPI_ISL_14823421 | A/Wild_geese/Hubei/H418/2021 | EPI_ISL_199230 | A/duck/Guangdong/04.22_DGCPLB018-O/2015 | EPI_ISL_4194 | A/Ck/YN/115/2004 |
| EPI_ISL_14823426 | A/Whooper_swan/Sanmenxia/Y6/2020 | EPI_ISL_199244 | A/goose/Guangdong/04.22_DGCP120-P/2015 | EPI_ISL_4396748 | A/Meleagris_gallopavo/Belgium/11293_001/2021 |
| EPI_ISL_14823549 | A/Whooper_swan/Sanmenxia/Y8/2020 | EPI_ISL_199262 | A/duck/Guangdong/04.23_DGQTXC228-O/2015 | EPI_ISL_4538 | A/blackbird/Hunan/1/2004 |
| EPI_ISL_14823646 | A/Whooper_swan/Sanmenxia/Y10/2020 | EPI_ISL_199268 | A/goose/Guangdong/04.22_DGCP118-P/2015 | EPI_ISL_4558108 | A/Sichuan/06689/2021 |
| EPI_ISL_14823672 | A/Whooper_swan/Sanmenxia/Y11/2020 | EPI_ISL_199290 | A/duck/Guangdong/04.23_DGQTXC236-P/2015 | EPI_ISL_4568642 | A/Hunan/09285/2021 |
| EPI_ISL_14823813 | A/Whooper_swan/Sanmenxia/Y14/2020 | EPI_ISL_199291 | A/duck/Guangdong/04.23_DGQTXC213-O/2015 | EPI_ISL_4568643 | A/Hunan/09911/2021 |
| EPI_ISL_14823863 | A/Whooper_swan/Sanmenxia/Y51/2020 | EPI_ISL_199407 | A/duck/Guangdong/03.26_DGCP077-O/2015 | EPI_ISL_4568644 | A/Chongqing/02/2021 |
| EPI_ISL_14824026 | A/Whooper_swan/Sanmenxia/G15/2020 | EPI_ISL_199441 | A/chicken/Guangxi/04.10_NM140-O/2015 | EPI_ISL_4808840 | A/mallard/Denmark/12947-12/2020-10-26 |
| EPI_ISL_14824091 | A/Whooper_swan/Sanmenxia/Y15/2020 | EPI_ISL_199444 | A/chicken/Guangxi/04.10_NM140/2015 | EPI_ISL_4852 | A/chicken/Henan/01/2004 |
| EPI_ISL_14824095 | A/Whooper_swan/Sanmenxia/Y16/2020 | EPI_ISL_199465 | A/pigeon/Guangdong/04.16_SZLGWL006/2015 | EPI_ISL_4853 | A/Chicken/Henan/210/2004 |
| EPI_ISL_14824122 | A/Whooper_swan/Sanmenxia/Y52/2020 | EPI_ISL_200238 | A/duck/Jiangxi/01.14_NCJD034-P/2015_Mixed | EPI_ISL_502069 | A/muscovy_duck/Japan/AQ-HE30-77C1/2018 |
| EPI_ISL_14835960 | A/Whooper_swan/Sanmenxia/H1/2020 | EPI_ISL_200240 | A/duck/Guangdong/04.23_DGQTSJ127-O/2015_Mixed | EPI_ISL_502202 | A/mink/Eastern_China/032/2018 |
| EPI_ISL_14835963 | A/Whooper_swan/Sanmenxia/H615/2020 | EPI_ISL_200242 | A/goose/Guangdong/04.23_DGQTXC237-O/2015_Mixed | EPI_ISL_502736 | A/Anas_platyrhynchos/Belgium/10402_H195386/2017 |
| EPI_ISL_14835965 | A/Whooper_swan/Sanmenxia/H810/2020 | EPI_ISL_200244 | A/duck/Guangdong/04.23_DGQTSJ124-O/2015_Mixed | EPI_ISL_503006 | A/mallard/Tottori/31C/2019 |
| EPI_ISL_14835968 | A/Whooper_swan/Sanmenxia/Y24/2020 | EPI_ISL_200257 | A/duck/Guangdong/03.27_DGQTSJ121-P/2015_Mixed | EPI_ISL_503015 | A/duck/Nha_Trang/271/2018 |
| EPI_ISL_14835974 | A/Whooper_swan/Sanmenxia/Y18/2020 | EPI_ISL_200258 | A/environment/Jiangsu/12.30_WZNHQ012/2014_Mixed | EPI_ISL_503599 | A/duck/Viet_Nam/HU12-970/2019 |
| EPI_ISL_14835976 | A/Whooper_swan/Sanmenxia/Y56/2020 | EPI_ISL_200259 | A/duck/Guangdong/04.15_SZBAXQ019/2015_Mixed | EPI_ISL_503819 | A/duck/Viet_Nam/HU12-1473/2019 |
| EPI_ISL_14836024 | A/Whooper_swan/Sanmenxia/Y19/2020 | EPI_ISL_200260 | A/environment/Guangdong/04.16_SZLGWLShui9/2015_Mixed | EPI_ISL_504984 | A/duck/Viet_Nam/HU13-161/2019 |
| EPI_ISL_14836025 | A/Whooper_swan/Sanmenxia/Y20/2020 | EPI_ISL_200272 | A/duck/Jiangxi/04.01_NCDZT0304-O/2015_Mixed | EPI_ISL_505073 | A/chicken/Sichuan/k141/2017 |
| EPI_ISL_14836026 | A/Whooper_swan/Sanmenxia/Y21/2020 | EPI_ISL_200275 | A/duck/Hubei/03.06__WHWTZ0049-P/2015_Mixed | EPI_ISL_505406 | A/chicken/AL_Suez/184/2018 |
| EPI_ISL_14836027 | A/Whooper_swan/Sanmenxia/Y23-1/2020 | EPI_ISL_200277 | A/duck/Hubei/03.06__WHWTZ0150-P/2015_Mixed | EPI_ISL_505432 | A/chicken/South_Africa/499723/2018 |
| EPI_ISL_14836078 | A/Whooper_swan/Sanmenxia/G27/2020 | EPI_ISL_200279 | A/duck/Hunan/03.09_YYFQH0036-P/2015_Mixed | EPI_ISL_5095555 | A/chicken/Germany-NI/AI00547/2021 |
| EPI_ISL_14836080 | A/Whooper_swan/Sanmenxia/G29/2020 | EPI_ISL_200282 | A/environment/Yunnan/03.17_DQJT0015-Z/2015_Mixed | EPI_ISL_5098142 | A/Hawaiian_goose/Germany-RP/AI00856/2021 |
| EPI_ISL_14836081 | A/Whooper_swan/Sanmenxia/Y26/2020 | EPI_ISL_200283 | A/chicken/Yunnan/03.16_DQXYL061-1-O/2015_Mixed | EPI_ISL_5098154 | A/turkey/Germany-MV/AI01127/2021 |
| EPI_ISL_14836082 | A/Whooper_swan/Sanmenxia/Y27/2020 | EPI_ISL_200284 | A/environment/Hunan/04.14_YYGK394/2015_Mixed | EPI_ISL_5099372 | A/Laridae/Germany-SH/AI01498/2021 |
| EPI_ISL_14836083 | A/Whooper_swan/Sanmenxia/Y31/2020 | EPI_ISL_200825 | A/duck/Yunnan/07.15_DQNPH129/2015 | EPI_ISL_5100056 | A/turkey/Germany-NI/AI02013/2021 |
| EPI_ISL_14836086 | A/Whooper_swan/Sanmenxia/Y36/2020 | EPI_ISL_201355 | A/duck/Fujian/05.07_FZHMDK14-O/2015 | EPI_ISL_5102130 | A/chicken/Germany-NW/AI02054/2021 |
| EPI_ISL_14836098 | A/Whooper_swan/Sanmenxia/Y54/2020 | EPI_ISL_201378 | A/duck/Jiangxi/09.04_JX-14-11/2014 | EPI_ISL_5115967 | A/chicken/Germany-SH/AI02312/2021 |
| EPI_ISL_14836101 | A/Whooper_swan/Sanmenxia/Y57/2020 | EPI_ISL_201799 | A/duck/Guangdong/s14044/2014 | EPI_ISL_5116063 | A/swan/Germany-HE/AI02335/2021 |
| EPI_ISL_14836369 | A/Whooper_swan/Sanmenxia/Y48B/2020 | EPI_ISL_202554 | A/chicken/Sichuan/J1/2014 | EPI_ISL_5159426 | A/Hunan/10117/2021 |
| EPI_ISL_14836371 | A/Whooper_swan/Sanmenxia/Y49/2020 | EPI_ISL_203734 | A/goose/Zhejiang/77167/2014 | EPI_ISL_5234711 | A/duck/Laos/NL-2175410/2021 |
| EPI_ISL_14836374 | A/Whooper_swan/Sanmenxia/G25/2020 | EPI_ISL_203736 | A/chicken/Zhejiang/727159/2014 | EPI_ISL_5234724 | A/duck/Laos/NL-2175411/2021 |
| EPI_ISL_14836376 | A/Whooper_swan/Sanmenxia/Y25/2020 | EPI_ISL_203741 | A/goose/Zhejiang/925036/2014 | EPI_ISL_5234816 | A/duck/Laos/NL-2175412/2021 |
| EPI_ISL_14836426 | A/Whooper_swan/Sanmenxia/B560/2020 | EPI_ISL_203742 | A/goose/Zhejiang/925105/2014 | EPI_ISL_525449 | A/domestic_duck/Poland/219/2020 |
| EPI_ISL_14836593 | A/Whooper_swan/Sanmenxia/B312/2020 | EPI_ISL_203745 | A/goose/Zhejiang/112080/2014 | EPI_ISL_5260417 | A/brown-headed_gull/Tibet/1-1/2021 |
| EPI_ISL_14840034 | A/Whooper_swan/Sanmenxia/B1770/2020 | EPI_ISL_203746 | A/goose/Zhejiang/1120132/2014 | EPI_ISL_5260419 | A/duck/Guangdong/S1269/2021 |
| EPI_ISL_14840053 | A/Whooper_swan/Sanmenxia/B2465/2021 | EPI_ISL_203750 | A/duck/Zhejiang/925019/2014 | EPI_ISL_5260420 | A/duck/Guangxi/S10099/2021 |
| EPI_ISL_14841913 | A/mallard/22P019377/France/2022 | EPI_ISL_205119 | A/duck/Wenzhou/YHQL22/2014 | EPI_ISL_5260421 | A/duck/Guangxi/S10263/2021 |
| EPI_ISL_14867042 | A/duck/Kagoshima/NIES229/2020 | EPI_ISL_205120 | A/duck/Wuhan/JXYFB22/2015 | EPI_ISL_5260422 | A/duck/Guangxi/S11043/2021 |
| EPI_ISL_14933724 | A/duck/France/22P020165/2022 | EPI_ISL_205121 | A/duck/Taizhou/TZYG12/2015 | EPI_ISL_5260426 | A/duck/Guangxi/S21194/2021 |
| EPI_ISL_14937000 | A/snow_goose/Kansas/W22-199D/2022 | EPI_ISL_205141 | A/turtledove/Wuhan/WHBJ26/2014 | EPI_ISL_5260445 | A/duck/Hebei/S1070/2021 |
| EPI_ISL_14937070 | A/bald_eagle/Florida/W22-195/2022 | EPI_ISL_205503 | A/Shenzhen/TH001/2015 | EPI_ISL_5260452 | A/green-winged_teal/Guangdong/SD004/2021 |
| EPI_ISL_14937098 | A/American_pelican/Kansas/W22-200/2022 | EPI_ISL_205856 | A/chicken/Yunnan/1/2014 | EPI_ISL_5260462 | A/whooper_swan/Shanxi/4-1/2020 |
| EPI_ISL_14937103 | A/bald_eagle/Georgia/W22-194A/2022 | EPI_ISL_205959 | A/Duck/Guangdong/SS1A1/2014H5N6 | EPI_ISL_5260463 | A/whooper_swan/Shanxi/4-2/2020 |
| EPI_ISL_14937105 | A/bald_eagle/Florida/W22-189/2022 | EPI_ISL_205964 | A/Chicken/Guangdong/FG594/2015H5N6 | EPI_ISL_5260466 | A/grebe/Shandong/SC184/2021 |
| EPI_ISL_150015 | A/Cambodia/X0810301/2013 | EPI_ISL_205966 | A/Goose/_Guangdong_/SSLBY/2015H5N6 | EPI_ISL_5463797 | A/duck/Saratov/29-02V/2021 |
| EPI_ISL_15004053 | A/Pekin_duck/Indiana/22-010611-001/2022 | EPI_ISL_205967 | A/Goose/_Guangdong_/SSZYP/2015H5N6 | EPI_ISL_576280 | A/duck/Guangdong/3231/2018 |
| EPI_ISL_15004060 | A/chicken/North_Dakota/22-010657-001/2022 | EPI_ISL_206036 | A/Shenzhen/1/2016 | EPI_ISL_576374 | A/duck/Guangdong/3111/2018 |
| EPI_ISL_15004062 | A/chicken/Colorado/22-010668-001/2022 | EPI_ISL_206568 | A/Guangdong/SZ872/2015H5N6 | EPI_ISL_576375 | A/goose/Guangdong/3451/2018 |
| EPI_ISL_15004376 | A/chicken/Minnesota/22-010928-001/2022 | EPI_ISL_206569 | A/Guangdong/ZQ874/2015 | EPI_ISL_576376 | A/goose/Guangdong/3441/2018 |
| EPI_ISL_15063427 | A/chicken/Ehime/TU10-2-13/2022 | EPI_ISL_208830 | A/goose/Eastern_China/L1214/2012 | EPI_ISL_576378 | A/duck/Guangdong/3311/2018 |
| EPI_ISL_15063429 | A/chicken/Kagoshima/TU2-18_19/2021 | EPI_ISL_208834 | A/duck/Eastern_China/S0131/2014 | EPI_ISL_576379 | A/goose/Guangdong/3241/2018 |
| EPI_ISL_15069398 | A/European_Herring_Gull/Netherlands/12/2022 | EPI_ISL_209131 | A/duck/Mongolia/543/2015 | EPI_ISL_576380 | A/goose/Guangdong/3452/2018 |
| EPI_ISL_15078237 | A/Canada_goose/Wyoming/22-011671-001/2022 | EPI_ISL_217941 | A/Anas_crecca/Hubei/Chenhu1623-5/2014_H5N6 | EPI_ISL_576381 | A/duck/Guangdong/3151/2018 |
| EPI_ISL_15078241 | A/bald_eagle/Wyoming/22-013015-001/2022 | EPI_ISL_219776 | A/Environment/Hunan/18478/2014 | EPI_ISL_5865695 | A/green-winged-teal/Georgia/DT-22246/2020 |
| EPI_ISL_15081424 | A/wigeon/Sakhalin/37M/2021 | EPI_ISL_219790 | A/Environment/Chongqing/45208/2015 | EPI_ISL_603133 | A/Eurasian_Wigeon/Netherlands/1/2020 |
| EPI_ISL_15088308 | A/Mallard/Netherlands/5/2022 | EPI_ISL_219808 | A/Environment/Guangdong/40113/2015 | EPI_ISL_625671 | A/whooper_swan/Inner_Mongolia/w1-1/2020 |
| EPI_ISL_151729 | A/duck/Bangladesh/19097/2013 | EPI_ISL_219828 | A/Guangdong/99710/2014 | EPI_ISL_625672 | A/mute_swan/Inner_Mongolia/w2-1/2020 |
| EPI_ISL_152085 | A/duck/Guangdong/wy11/2008 | EPI_ISL_219874 | A/duck/Taiwan/A3400/2015 | EPI_ISL_63022 | A/duck/Hunan/8/2008 |
| EPI_ISL_15234656 | A/bearded_vulture/Spain/2116-3_22VIR8632-8/2022 | EPI_ISL_221706 | A/duck/Guangdong/01.01_SZSGXJK001-G/2016 | EPI_ISL_632316 | A/eurasian_curlew/Netherlands/20016890-001/2020 |
| EPI_ISL_15350905 | A/chicken/Ghana/AVL-763_21VIR7050-39/2021 | EPI_ISL_221710 | A/duck/Guangdong/01.01_SZSGXJK004-Y/2016 | EPI_ISL_63471 | A/chicken/Zhejiang/HJ/2007 |
| EPI_ISL_153620 | A/chicken/China/AH/2012 | EPI_ISL_221712 | A/duck/Guangdong/01.01_SZSGXJK005-Y/2016 | EPI_ISL_644123 | A/turkey/Omsk/0003/2020 |
| EPI_ISL_15430209 | A/common_crane/Yunnan-Huize/11/2021 | EPI_ISL_221714 | A/duck/Guangdong/01.01_SZSGXJK006-Y/2016 | EPI_ISL_644125 | A/goose/Omsk/0111/2020 |
| EPI_ISL_15433349 | A/common_crane_/Yunnan-Huize/22/2021 | EPI_ISL_221716 | A/duck/Guangdong/01.01_SZSGXJK007-G/2016 | EPI_ISL_644127 | A/goose/Omsk/0113/2020 |
| EPI_ISL_15435500 | A/common_crane/Yunnan-Huize/24/2021 | EPI_ISL_221748 | A/water/Hunan/140/2014 | EPI_ISL_644139 | A/goose/Omsk/0071/2020 |
| EPI_ISL_15435511 | A/common_crane/Yunnan-Huize/27/2021 | EPI_ISL_2227278 | A/goose/Netherlands/21028502-002/21028502/2021 | EPI_ISL_644141 | A/goose/Omsk/0074/2020 |
| EPI_ISL_15585751 | A/Chicken/England/128321/2022 | EPI_ISL_223928 | A/chicken/Ghana/20/2015 | EPI_ISL_644150 | A/chicken/Omsk/0112/2020 |
| EPI_ISL_15585900 | A/herring_gull/England/324803/2022 | EPI_ISL_224580 | A/great_crested_grebe/Uvs-Nuur_Lake/341/2016 | EPI_ISL_644155 | A/chicken/Omsk/0119/2020 |
| EPI_ISL_15613574 | A/Guangdong/1/2021 | EPI_ISL_2246 | A/Chicken/Hong_Kong/YU562/01 | EPI_ISL_644158 | A/chicken/Omsk/0073/2020 |
| EPI_ISL_15618 | A/duck/Shantou/4912/2001 | EPI_ISL_224732 | A/Bar-headed_Goose/Qinghai/BTY14-LU/2016 | EPI_ISL_64823 | A/chicken/Dairi/BPPVI/2005 |
| EPI_ISL_15647834 | A/duck/Korea/H493/2022 | EPI_ISL_224879 | A/duck/Zhejiang/6DK19/2013 | EPI_ISL_64956 | A/whooper_swan/Mongolia/244/2005 |
| EPI_ISL_15648 | A/duck/Hunan/795/2002 | EPI_ISL_2250 | A/Silky_Chicken/Hong_Kong/SF189/01 | EPI_ISL_64958 | A/Bar_headed_goose/Qinghai/1A/2005 |
| EPI_ISL_15679 | A/duck/Yunnan/426/2004 | EPI_ISL_2251 | A/Quail/Hong_Kong/SF203/01 | EPI_ISL_65330 | A/swine/Anhui/ca/2004 |
| EPI_ISL_15723338 | A/Environment/Hunan/10170/2016 | EPI_ISL_230759 | A/muscovy_duck/Vietnam/LBM823/2015 | EPI_ISL_654848 | A/chicken/Nghe_An/842VTC/2020 |
| EPI_ISL_15723339 | A/Environment/Hunan/11041/2016 | EPI_ISL_232493 | A/Egypt/N04915/2014__NIBRG-306 | EPI_ISL_654859 | A/chicken/Thanh_Hoa/1152VTC/2020 |
| EPI_ISL_15723481 | A/Environment/Guangxi/24894/2018 | EPI_ISL_234670 | A/Copsychus_saularis/Guangdong/SW8/2014 | EPI_ISL_660264 | A/Gallus_gallus/Belgium/12168_002/2020 |
| EPI_ISL_15723482 | A/Environment/Guangxi/24886/2018 | EPI_ISL_234671 | A/Gallinula_chloropus/Guangdong/GZ174/2014 | EPI_ISL_661313 | A/Anser_albifrons/Belgium/11956_005/2020 |
| EPI_ISL_15723485 | A/Environment/Guangdong/27940/2018 | EPI_ISL_234919 | A/duck/France/150233/2015 | EPI_ISL_67388 | A/chicken/Korea/es/2003 |
| EPI_ISL_15723492 | A/Environment/Guangdong/30702/2018 | EPI_ISL_234920 | A/duck/France/150236/2015 | EPI_ISL_6757629 | A/Duck/Guangdong/21314/2021 |
| EPI_ISL_15723507 | A/Environment/Guangxi/33095/2018 | EPI_ISL_235740 | A/mallard/Alaska/AH0088535/2016 | EPI_ISL_6757630 | A/Goose/Sichuan/21406-5/2021 |
| EPI_ISL_15723525 | A/Environment/Guangxi/01878/2018 | EPI_ISL_237554 | A/painted_stork/India/10CA03/2016 | EPI_ISL_6757631 | A/Duck/Sichuan/21406-8/2021 |
| EPI_ISL_15723894 | A/Environment/Guangxi/01885/2018 | EPI_ISL_237921 | A/wild_duck/Poland/82A/2016 | EPI_ISL_6760679 | A/Duck/Sichuan/21826-5/2021 |
| EPI_ISL_15723897 | A/Environment/Guangxi/09150/2019 | EPI_ISL_237944 | A/tufted_duck/Germany/AR8444-L01987/2016 | EPI_ISL_6760686 | A/Chicken/Hebei/211021/2021_Mixed |
| EPI_ISL_15723898 | A/Environment/Hubei/43318/2019 | EPI_ISL_237952 | A/chicken/Japan/AnimalQuarantine-HE144/2016 | EPI_ISL_6760687 | A/Goose/Guangdong/211057/2021 |
| EPI_ISL_15723901 | A/Environment/Guangdong/34076/2019 | EPI_ISL_237953 | A/duck/Tsukuba/922/08 | EPI_ISL_6772736 | A/Chicken/Guangdong/211064-1/2021 |
| EPI_ISL_15723904 | A/Environment/Guangxi/25038/2019 | EPI_ISL_238016 | A/whistling_swan/Shimane/42/80 | EPI_ISL_6772737 | A/Chicken/Guangdong/211064-2/2021 |
| EPI_ISL_15723906 | A/Environment/Guangxi/32383/2019 | EPI_ISL_238017 | A/duck/Chiba/1/2010 | EPI_ISL_6772738 | A/Chicken/Guangdong/211064-3/2021 |
| EPI_ISL_15723909 | A/Environment/Guangxi/45278/2019 | EPI_ISL_238023 | A/duck/Tsukuba/30/07 | EPI_ISL_6772739 | A/Chicken/Guangdong/211064-4/2021 |
| EPI_ISL_15723912 | A/Environment/Guangxi/39500/2019 | EPI_ISL_238025 | A/duck/Tsukuba/394/2005 | EPI_ISL_6772759 | A/Chicken/Guangdong/211064-5/2021 |
| EPI_ISL_15723916 | A/Environment/Guangdong/09210/2019 | EPI_ISL_238028 | A/pochard/Fukui/131816/2013 | EPI_ISL_6772898 | A/Goose/Guangdong/211106-2/2021 |
| EPI_ISL_15723922 | A/Environment/Guangdong/09230/2019 | EPI_ISL_239260 | A/chicken/Korea/H23/2016 | EPI_ISL_6780643 | A/Duck/Luxor/51/2018 |
| EPI_ISL_15723925 | A/Environment/Guangdong/14024/2020 | EPI_ISL_239271 | A/spot_billed_duck/Korea/WB141/2016 | EPI_ISL_6780663 | A/chicken/Luxor/103/2018 |
| EPI_ISL_15812579 | A/Anser_anser_domesticus/Belgium/10918_0002/2022 | EPI_ISL_239351 | A/duck/Hyogo/1/2016 | EPI_ISL_6781352 | A/goose/China/Wuhu01/2019 |
| EPI_ISL_15837851 | A/Cape_cormorant/South_Africa/21100176B/2021 | EPI_ISL_239387 | A/duck/Toyama/161010/2016 | EPI_ISL_6795237 | A/duck/China/FJ1543/2015 |
| EPI_ISL_15854273 | A/chicken/Foshan/G817/2019 | EPI_ISL_239391 | A/duck/Fukui/181019/2016 | EPI_ISL_6795249 | A/duck/China/FJ19179/2017 |
| EPI_ISL_15957648 | A/chicken/England/138155/2022 | EPI_ISL_239399 | A/duck/Aichi/231019/2016 | EPI_ISL_6795250 | A/duck/China/JX1519/2015 |
| EPI_ISL_15957972 | A/turkey/England/150421/2022 | EPI_ISL_240012 | A/duck/France/161108h/2016 | EPI_ISL_6795257 | A/duck/China/FJ18248/2019 |
| EPI_ISL_15957978 | A/chicken/England/151023/2022 | EPI_ISL_240102 | A/domestic_goose/Poland/33/2016 | EPI_ISL_6795261 | A/duck/China/FJ1829/2019 |
| EPI_ISL_16157545 | A/chicken/Ecuador/02/2022 | EPI_ISL_240109 | A/chicken/Kalmykia/2661/2016 | EPI_ISL_6795264 | A/duck/China/FJ1904/2019 |
| EPI_ISL_16166662 | A/wild_duck/Shandong/W6252/2019 | EPI_ISL_240528 | A/chicken/Niigata/1-2C/2016 | EPI_ISL_6795267 | A/duck/China/FJ1921/2019 |
| EPI_ISL_16173542 | A/Chicken/ON/FAV-0208-133/2022 | EPI_ISL_240529 | A/muscovy_duck/Aomori/1-3T/2016s | EPI_ISL_6795268 | A/duck/China/FJ1922/2019 |
| EPI_ISL_16189981 | A/Franklin_s_Gull/AB/FAV-0505-51/2022 | EPI_ISL_240599 | A/chicken/Hokkaido/1-3-7T/2016 | EPI_ISL_6795270 | A/duck/China/FJ1931/2019 |
| EPI_ISL_16190673 | A/Chicken/AB/FAV-0832/2022 | EPI_ISL_240608 | A/black_headed_gull/Ibaraki/258T/2016 | EPI_ISL_6795271 | A/duck/China/FJ19323/2019 |
| EPI_ISL_16199040 | A/chicken/Austria/2514-19_22VIR11382-1/2022 | EPI_ISL_240610 | A/duck/Ibaraki/102/2016 | EPI_ISL_6795272 | A/duck/China/FJ19332/2019 |
| EPI_ISL_16208969 | A/tundra_swan/Shanghai/CM20111601/2020 | EPI_ISL_240703 | A/Hunan/55555/2016 | EPI_ISL_6795278 | A/Mule_Duck/China/FJ17152/2017 |
| EPI_ISL_16208999 | A/Guangdong/lgf/2021 | EPI_ISL_240704 | A/Guangxi/55726/2016 | EPI_ISL_6829533 | A/chicken/Kagoshima/21A6T/2021 |
| EPI_ISL_16209000 | A/chicken/Egypt/V1345/2019 | EPI_ISL_240892 | A/turkey/Germany-NI/R10523/2016 | EPI_ISL_683751 | A/Eurasian_wigeon/Italy/20VIR7301-31/2020 |
| EPI_ISL_16209011 | A/chicken/Egypt/AL6/2019 | EPI_ISL_241745 | A/chicken/Kumamoto/1-7T/2016 | EPI_ISL_683997 | A/mute_swan/Wales/048069/2020 |
| EPI_ISL_16209012 | A/chicken/Egypt/AL2/2019 | EPI_ISL_241757 | A/black_swan/Ibaraki/256C/2016 | EPI_ISL_683999 | A/mute_swan/Wales/048068/2020 |
| EPI_ISL_16215736 | A/chicken/China/JM01/2020 | EPI_ISL_242404 | A/chicken/Gifu/1-10C/2017 | EPI_ISL_6959592 | A/mandarin_duck/Korea/WA585/2021 |
| EPI_ISL_16240456 | A/chicken/Idaho/22-014425-002-original/2022 | EPI_ISL_243344 | A/wild_pigeon/Jilin/CC01/2014 | EPI_ISL_696572 | A/chicken/Anhui/11.29_YHZGS007-O/2018 |
| EPI_ISL_16249 | A/duck/Vietnam/53/2007 | EPI_ISL_243680 | A/chicken/Miyazaki/2-1T/2017 | EPI_ISL_696991 | A/goose/Fujian/3.15_FZHX0001-O/2018 |
| EPI_ISL_16271855 | A/domestic_goose/Tennessee/22-036130-001-original/2022 | EPI_ISL_244486 | A/chicken/Vietnam/NCVD14-A324/2014 | EPI_ISL_696992 | A/goose/Fujian/3.15_FZHX0007-O/2018 |
| EPI_ISL_16297127 | A/turkey/Minnesota/22-034666-001-original/2022 | EPI_ISL_244514 | A/goose/Vietnam/NCVD-15A27/2015 | EPI_ISL_696993 | A/goose/Fujian/3.15_FZHX0010-O/2018 |
| EPI_ISL_16314941 | A/chicken/Guangdong/18/2018 | EPI_ISL_244518 | A/chicken/Vietnam/NCVD-15A59/2015 | EPI_ISL_697002 | A/duck/Guangdong/7.20_DGCP049-C/2017 |
| EPI_ISL_16314943 | A/chicken/Guangdong/20/2018 | EPI_ISL_244526 | A/duck/Hubei/ZYSYG18/2015 | EPI_ISL_697098 | A/chicken/Yunnan/12.22_DQXBL001-O/2018 |
| EPI_ISL_163368_and_EPI_ISL_139735 | A/chicken/Indonesia/BL/2003 | EPI_ISL_244527 | A/duck/Hubei/ZYSYG5/2015 | EPI_ISL_697099 | A/chicken/Yunnan/12.22_DQXBL001-C/2018 |
| EPI_ISL_163493 | A/Sichuan/26221/2014 | EPI_ISL_244534 | A/chicken/Taishun/TS2/2016 | EPI_ISL_697110 | A/duck/Zhejiang/05.11_HZBX011-O/2018 |
| EPI_ISL_16384153 | A/Chicken/England/159386/2022 | EPI_ISL_244537 | A/chicken/Ganzhou/GZ50/2015 | EPI_ISL_697189 | A/duck/Fujian/11.09_FZHX-O/2017 |
| EPI_ISL_16384156 | A/Domestic_duck/England/159770/2022 | EPI_ISL_24603 | A/Anhui/1/2005 | EPI_ISL_697352 | A/chicken/Jiangxi/3.23_NCDZT15M3-OC/2018 |
| EPI_ISL_16384158 | A/Domestic_Goose/England/161197/2022 | EPI_ISL_24608 | A/Guangxi/1/2005 | EPI_ISL_697355 | A/chicken/Jiangxi/3.23_NCDZT63M3-OC/2018 |
| EPI_ISL_16384163 | A/domestic_goose/England/169312/2022 | EPI_ISL_247406 | A/black_headed_gull/Ibaraki/194T/2016 | EPI_ISL_697361 | A/chicken/Jiangxi/4.25_NCNP6Q3-OC/2018 |
| EPI_ISL_16443924 | A/domestic_duck/England/167913/2022 | EPI_ISL_24868 | A/Cambodia/R0405050/2007 | EPI_ISL_697363 | A/chicken/Jiangxi/6.21_NCDZT9S3-OC/2018 |
| EPI_ISL_16466440 | A/Changsha/1/2022 | EPI_ISL_24874 | A/Egypt/3300-NAMRU3/2008 | EPI_ISL_697390 | A/duck/Hunan/5.29_YYGK88P3-OC/2018 |
| EPI_ISL_16574507 | A/chicken/England/172702/2022 | EPI_ISL_249691 | A/chicken/Germany-MV/R10048/2016 | EPI_ISL_697412 | A/chicken/Hunan/10.27_YYGK55B4-OC/2018 |
| EPI_ISL_16574516 | A/chicken/England/172713/2022 | EPI_ISL_249692 | A/chicken/Germany-NI/R11406/2016 | EPI_ISL_697440 | A/chicken/Anhui/2.22_YHZGS013-O/2019 |
| EPI_ISL_16574544 | A/white-fronted_goose/Scotland/173468/2022 | EPI_ISL_252419 | A/guinea_fowl/France/150207n/2015 | EPI_ISL_697442 | A/chicken/Anhui/2.22_YHZGS022-C/2019 |
| EPI_ISL_16574586 | A/common_buzzard/England/255858/2022 | EPI_ISL_252830 | A/chicken/Ganzhou/GZ157/2016 | EPI_ISL_697446 | A/chicken/Shanxi/2.22_TGRL001-O/2019 |
| EPI_ISL_16574612 | A/black-headed_gull/England/120011/2022 | EPI_ISL_252831 | A/chicken/Ganzhou/GZ43/2016 | EPI_ISL_697450 | A/chicken/Shandong/2.25_TAWL001-O/2019 |
| EPI_ISL_16574710 | A/common_buzzard/England/396209/2022 | EPI_ISL_252834 | A/duck/Ganzhou/GZ151/2016 | EPI_ISL_697451 | A/chicken/Shandong/2.25_TAWL003-O/2019 |
| EPI_ISL_16574747 | A/greylag_goose/England/396600/2022 | EPI_ISL_253946 | A/chicken/Japan/AQ-HE144/2015 | EPI_ISL_697689 | A/duck/Mongolia/217/2018 |
| EPI_ISL_16613711 | A/chicken/Czech_Republic/67_orig/2023 | EPI_ISL_253949 | A/duck/Japan/AQ-HE72/2015 | EPI_ISL_697808 | A/chicken/Fujian/11.23_FZHX0006-O/2017 |
| EPI_ISL_16641771 | A/american_crow/MA/22HP00148/2022 | EPI_ISL_254745 | A/mallard_duck/Korea/WA137/2017 | EPI_ISL_697819 | A/chicken/Guizhou/12.20_ZYLJJ017-O/2017 |
| EPI_ISL_1665248 | A/chicken/Italy/21VIR1293-9/2021 | EPI_ISL_255182 | A/turkey/Italy/17VIR538-1/2017 | EPI_ISL_697929 | A/duck/Zhejiang/1026-HZBX001-C/2018 |
| EPI_ISL_166693 | A/duck/Beijing/FS01/2014 | EPI_ISL_255194 | A/Goose/Hungary/1030/2017 | EPI_ISL_698000 | A/chicken/Guangdong/7.20_DGCP050-O/2017 |
| EPI_ISL_166694 | A/duck/Beijing/CT01/2014 | EPI_ISL_255205 | A/Chicken/Hungary/2496/2017 | EPI_ISL_698027 | A/chicken/Jiangxi/6.21_NCDZT7S3-OC/2018 |
| EPI_ISL_16676477 | A/Anser_anser/Belgium/00939-0011/2022 | EPI_ISL_255213 | A/GuineaFowl/Hungary/596/2017 | EPI_ISL_698048 | A/duck/Hunan/4.26_YYGK85R3-OC/2018 |
| EPI_ISL_16718465 | A/hooded_crane/Kagoshima/KU-d89/2021_ | EPI_ISL_255469 | A/chicken/Hunan/HN40/2015 | EPI_ISL_698049 | A/duck/Hunan/4.26_YYGK88R3-OC/2018 |
| EPI_ISL_16810698 | A/Bubo_bubo/Belgium/09467_0001/2022 | EPI_ISL_255470 | A/chicken/Hunan/HN58/2015 | EPI_ISL_698050 | A/duck/Hunan/4.26_YYGK90R3-OC/2018 |
| EPI_ISL_16811189 | A/turkey/England/006787/2023 | EPI_ISL_255492 | A/duck/Hunan/193/2014 | EPI_ISL_698055 | A/duck/Hunan/5.29_YYGK90P3-OC/2018 |
| EPI_ISL_16811197 | A/chicken/England/010290/2023 | EPI_ISL_255529 | A/duck/Hunan/HN13/2015 | EPI_ISL_698056 | A/duck/Hunan/5.29_YYGK72P3-OC/2018 |
| EPI_ISL_16839065 | A/Cormorant/Namibia/141/2022 | EPI_ISL_2555532 | A/Cygnus_columbianus/Hubei/116/2020 | EPI_ISL_698057 | A/duck/Hunan/5.29_YYGK71P3-OC/2018 |
| EPI_ISL_16840595 | A/Chicken/China/30/2019 | EPI_ISL_2555534 | A/Cygnus_columbianus/Hubei/56/2020 | EPI_ISL_698062 | A/duck/Hunan/12.24_YYGK88E4-C/2018 |
| EPI_ISL_16840596 | A/Chicken/China/31/2019 | EPI_ISL_2555536 | A/Chlidonias_hybrida/Hubei/55/2020 | EPI_ISL_699166 | A/chicken/Anhui/8.28_YHZGS017-O/2018 |
| EPI_ISL_16840599 | A/Duck/Foshan/11/2019 | EPI_ISL_2555538 | A/Cygnus_columbianus/Hubei/53/2020 | EPI_ISL_699297 | A/chicken/Guangdong/12.29_SZBJ009-O/2016 |
| EPI_ISL_16868590 | A/environment/China/39187/2020 | EPI_ISL_2555541 | A/Cygnus_columbianus/Hubei/52/2020 | EPI_ISL_699298 | A/chicken/Guangdong/12.29_SZBJ010-O/2016 |
| EPI_ISL_16868591 | A/Duck/China/69361/2020 | EPI_ISL_2555542 | A/Cygnus_columbianus/Hubei/51/2020 | EPI_ISL_699346 | A/chicken/Guangdong/7.20_DGCP022-O/2017 |
| EPI_ISL_16886862 | A/Rattus_norvegicus/China/FS21/2021 | EPI_ISL_2555543 | A/Cygnus_columbianus/Hubei/50/2020 | EPI_ISL_699451 | A/chicken/Hunan/12.27_YYGK55J2-O/2016 |
| EPI_ISL_169422 | A/chicken/Miyazaki/3/2014 | EPI_ISL_2556113 | A/Cygnus_columbianus/Hubei/49/2020 | EPI_ISL_700932 | A/wild_bird/China/Y13/2019 |
| EPI_ISL_16955799 | A/large-billed_crow/Miyagi/0411G001/2022 | EPI_ISL_255689 | A/duck/Hunan/HN214/2015 | EPI_ISL_7049600 | A/bean_goose/Sweden/SVA211111SZ0372/FB004482/2021 |
| EPI_ISL_16956354 | A/Greylag_Goose/Scotland/015635/2023 | EPI_ISL_255752 | A/duck/Hunan/HN285/2015 | EPI_ISL_707016 | A/Whooper_swan/Mongolia/25/2020 |
| EPI_ISL_16969373 | A/duck/Arizona/S1-AZMCP12122022-S4/2022 | EPI_ISL_255758 | A/duck/Hunan/HN335/2015 | EPI_ISL_707453 | A/duck/Guangdong/11.18_SZBJ003-C/2016 |
| EPI_ISL_1697190 | A/peacock/Czech_Republic/6529-2/2021 | EPI_ISL_255761 | A/duck/Hunan/HN341/2015 | EPI_ISL_707454 | A/duck/Guangdong/11.18_SZBJ003-O/2016 |
| EPI_ISL_17051137 | A/turkey/BC/AIVPHL-130/2022 | EPI_ISL_255817 | A/duck/Hunan/HN74/2015 | EPI_ISL_707455 | A/duck/Guangdong/11.18_SZBJ007-O/2016 |
| EPI_ISL_17051399 | A/canada_goose/BC/AIVPHL-227/2022 | EPI_ISL_255840 | A/goose/Hunan/HN314/2015 | EPI_ISL_707456 | A/duck/Guangdong/7.20_DGCP015-C/2017 |
| EPI_ISL_17051410 | A/canada_goose/BC/AIVPHL-241/2022 | EPI_ISL_255846 | A/goose/Hunan/HN325/2015 | EPI_ISL_707475 | A/duck/Hunan/01.12_YYGK82H3-OC/2018 |
| EPI_ISL_17051431 | A/snow_goose/BC/AIVPHL-307/2022 | EPI_ISL_255850 | A/goose/SiChuan/15/2015 | EPI_ISL_707478 | A/duck/Hunan/03.24_YYGK174M2-C/2017 |
| EPI_ISL_17051476 | A/glaucous-winged_gull/BC/AIVPHL-356/2023 | EPI_ISL_255861 | A/muscovy/duck/Hunan/183/2014 | EPI_ISL_707483 | A/duck/Hunan/05.29_YYGK69P3-OC/2018 |
| EPI_ISL_17071926 | A/Anser_anser/Belgium/12540_0001/2022 | EPI_ISL_255865 | A/muscovy/duck/Hunan/232/2014 | EPI_ISL_707484 | A/duck/Hunan/05.29_YYGK80P3-OC/2018 |
| EPI_ISL_17072173 | A/Gallus_gallus/Belgium/01111_0002/2023 | EPI_ISL_255875 | A/muscovy/duck/Hunan/HN349/2015 | EPI_ISL_707489 | A/duck/Hunan/1.12_YYGK68H3-OC/2018 |
| EPI_ISL_17072782 | A/Greylag_Goose/England/175256/2022 | EPI_ISL_255878 | A/muscovy/duck/Hunan/HN352/2015 | EPI_ISL_707491 | A/duck/Hunan/1.12_YYGK74H3-OC/2018 |
| EPI_ISL_17075747 | A/Jiangsu/NJ210/2023 | EPI_ISL_255933 | A/Cormorant/Hungary/6102/2017 | EPI_ISL_707492 | A/duck/Hunan/1.12_YYGK98H3-OC/2018 |
| EPI_ISL_17090181 | A/duck/Vietnam/QN3253/2016 | EPI_ISL_256213 | A/Hubei/29578/2016 | EPI_ISL_707501 | A/duck/Hunan/11.30_YYGK62E3-OC/2017 |
| EPI_ISL_17104293 | A/Environment/Guangdong/08966/2019 | EPI_ISL_256462 | A/Mute_swan/Hungary/5879/2017 | EPI_ISL_707502 | A/duck/Hunan/11.30_YYGK63E3-OC/2017 |
| EPI_ISL_17104294 | A/Environment/Guangdong/08965/2019 | EPI_ISL_25690 | A/common_magpie/Hong_Kong/5052/2007 | EPI_ISL_707531 | A/duck/Hunan/6.27_YYGK79S2-OC/2017 |
| EPI_ISL_17104309 | A/Environment/Guangdong/34335/2019 | EPI_ISL_259923 | A/duck/Guangdong/673/2014 | EPI_ISL_707533 | A/duck/Jiangxi/01.11_NCNP101G2-OC/2017 |
| EPI_ISL_17104315 | A/Environment/Guangdong/27210/2019 | EPI_ISL_259924 | A/goose/Guangdong/674/2014 | EPI_ISL_707546 | A/duck/Jiangxi/11.29_NCDZT71H2-O/2016 |
| EPI_ISL_17104327 | A/Environment/Guangdong/26575/2018 | EPI_ISL_260059 | A/cormorant/Germany-SH/R896/2017 | EPI_ISL_707555 | A/duck/Jiangxi/2.28_NCNP25K3-OC/2018 |
| EPI_ISL_17104328 | A/Environment/Guangdong/26570/2018 | EPI_ISL_262 | A/chicken/Yamaguchi/7/2004 | EPI_ISL_707652 | A/goose/Fujian/11.3_FZHX1102-C/2016 |
| EPI_ISL_17104329 | A/Environment/Guangdong/26566/2018 | EPI_ISL_262056 | A/greylag_goose/Germany-NI/AR11353-L02142/2016 | EPI_ISL_707653 | A/goose/Fujian/3.15_FZHX0008-C/2018 |
| EPI_ISL_17104330 | A/Environment/Guangdong/26564/2018 | EPI_ISL_262488 | A/duck/Chiba/51/07 | EPI_ISL_707658 | A/goose/Guangdong/7.20_DGCP010-C/2017 |
| EPI_ISL_17104333 | A/Environment/Guangdong/06083/2018 | EPI_ISL_266 | A/crow/Osaka/102/2004 | EPI_ISL_710505 | A/turkey/England/038115/2020 |
| EPI_ISL_17104334 | A/Environment/Zhejiang/05834/2018 | EPI_ISL_266596 | A/swine/Guangdong/G3/2015 | EPI_ISL_710507 | A/Greylag_goose/England/032698/2020 |
| EPI_ISL_17104335 | A/Environment/Guangxi/05711/2018 | EPI_ISL_266820 | A/Bean_goose/Hubei/CH-i122/2017_H5N8 | EPI_ISL_718266 | A/Jiangsu/1/2020 |
| EPI_ISL_17104336 | A/Environment/Guangxi/05706/2018 | EPI_ISL_2685846 | A/tundra_swan/Hubei/BQ3/2020 | EPI_ISL_7267244 | A/common_buzzard/Netherlands/21038793-001/2021 |
| EPI_ISL_17104351 | A/Environment/Hainan/31766/2018 | EPI_ISL_2685848 | A/tundra_swan/Hubei/BQ6/2020 | EPI_ISL_7267254 | A/greylag_goose/Netherlands/21039376-001/2021 |
| EPI_ISL_17104462 | A/Environment/Hunan/25036/2018 | EPI_ISL_268620 | A/Buzzard/NL-Durgerdam/16015100-004/2016 | EPI_ISL_73305 | A/Guangxi/1/2008 |
| EPI_ISL_17104468 | A/Environment/Guangdong/29711/2018 | EPI_ISL_268634 | A/Dk/NL-Stolwijk/16016291-016-020/2016 | EPI_ISL_7379464 | A/Duck/Sichuan/21044-2/2021 |
| EPI_ISL_17104469 | A/Environment/Guangdong/29652/2018 | EPI_ISL_268938 | A/mute_swan/Czech_Republic/967-17/2017 | EPI_ISL_7379465 | A/Goose/Henan/21056-2/2021 |
| EPI_ISL_17104470 | A/Environment/Guangdong/29648/2018 | EPI_ISL_2713170 | A/tundra_swan/Hubei/BQ9/2020 | EPI_ISL_7379509 | A/Duck/Guangdong/21057/2021 |
| EPI_ISL_17104471 | A/Environment/Guangdong/29646/2018 | EPI_ISL_2713372 | A/bean_goose/Hubei/BQ11/2020 | EPI_ISL_7380253 | A/Goose/Jiangsu/21153-2/2021 |
| EPI_ISL_17104472 | A/Environment/Guangdong/29644/2018 | EPI_ISL_277041 | A/duck/Wuxi/7249/2015 | EPI_ISL_7380500 | A/Goose/Shandong/21153-3/2021 |
| EPI_ISL_17104474 | A/Environment/Hunan/28868/2018 | EPI_ISL_278026 | A/Chicken/Huizhou/16274/2016 | EPI_ISL_738051 | A/chicken/Kagawa/L7T/2020 |
| EPI_ISL_17104476 | A/Environment/Guangdong/27826/2018 | EPI_ISL_278031 | A/Quail/Zhanjiang/16887/2016 | EPI_ISL_7380623 | A/Duck/Shandong/21232-5/2021 |
| EPI_ISL_17104480 | A/Environment/Guangxi/40878/2017 | EPI_ISL_278040 | A/peregrine_falcon/HK/4955/2015 | EPI_ISL_7380813 | A/Duck/Guangdong/21316/2021 |
| EPI_ISL_17104481 | A/Environment/Guangxi/40954/2017 | EPI_ISL_279038 | A/crane/Kagoshima/KU-48/2016 | EPI_ISL_7381026 | A/Chicken/Liaoning/21346-2/2021 |
| EPI_ISL_171655 | A/waterfowl/Korea/S005/2014 | EPI_ISL_280674 | A/mute_swan/Switzerland/V0244.2-L02307/2017 | EPI_ISL_7381115 | A/Goose/Liaoning/21640/2021 |
| EPI_ISL_17179654 | A/heron/Switzerland-Basel/230080/2023 | EPI_ISL_2825365 | A/duck/Guangdong/SE0468/2018 | EPI_ISL_7381438 | A/Duck/Guangdong/21964/2021 |
| EPI_ISL_17214735 | A/pink-footed_goose/England/398366/2023 | EPI_ISL_283704 | A/Duck/Yunnan/YN-4/2015__ | EPI_ISL_7381695 | A/Goose/Guangdong/211030-1/2021 |
| EPI_ISL_17257530 | A/chicken/England/038958/2023 | EPI_ISL_283705 | A/Chicken/Yunnan/YN-7/2016 | EPI_ISL_74313 | A/Egypt/N03072/2010 |
| EPI_ISL_17261964 | A/Env/Fujian/C19035050/2019 | EPI_ISL_283967 | A/Chicken/Yunnan/YN-10/2015 | EPI_ISL_7457654 | A/Chicken/Guangdong/211106-3/2021 |
| EPI_ISL_17262174 | A/Env/Fujian/C19209014/2019 | EPI_ISL_283970 | A/Ostrich/Guangxi/GX-1/2017 | EPI_ISL_7591058 | A/Anas_platyrhynchos_domestica/Belgium/5517/2021 |
| EPI_ISL_17267157 | A/European_herring_gull/Netherlands/9/2023 | EPI_ISL_284650 | A/Anhui/33162/2016 | EPI_ISL_7591585 | A/Anas_platyrhynchos/Belgium/827/2020 |
| EPI_ISL_173146 | A/duck/Vietnam/LBM360c1-4-1/2013 | EPI_ISL_284683 | A/Ostrich/South_Africa/S2017/08_0268_P2/2017 | EPI_ISL_759851 | A/chicken/Chiba/2T/2020 |
| EPI_ISL_173478 | A/duck/Jiangxi/13469/2014 | EPI_ISL_285509 | A/Ostrich/South_Africa/S2017/08_0362_P11/2017 | EPI_ISL_7622863 | A/Anas_platyrhynchos/Belgium/204_0003/2020 |
| EPI_ISL_17353838 | A/chicken/Magdalena/ICA-3503/2022 | EPI_ISL_285621 | A/chicken/Egypt/Gharbiya-15/2017 | EPI_ISL_7623789 | A/Gallus_gallus/Belgium/9247/2021 |
| EPI_ISL_17371282 | A/duck/Hulu_Sungai_Utara/A0522064-06/2022 | EPI_ISL_28830 | A/chicken/Vietnam/NCVD-016/2008 | EPI_ISL_76680 | A/Vietnam/HN31388M1/2007 |
| EPI_ISL_17371283 | A/duck/Hulu_Sungai_Utara/A0522064-03-04/2022 | EPI_ISL_288362 | A/chicken/Greece/39_2017/2017 | EPI_ISL_76684 | A/Vietnam/HN31432M/2008 |
| EPI_ISL_17373074 | A/duck/North_Carolina/W22-1114A/2022 | EPI_ISL_292185 | A/Great_Cormorant/Qinghai/Y01/2016 | EPI_ISL_7778761 | A/mute_swan/Estonia/TA2108545-1_21VIR7512-5/2021 |
| EPI_ISL_173878 | A/gyrfalcon/Washington/41088-6/2014 | EPI_ISL_292196 | A/Bar-headed_Goose/Qinghai/a32/2016 | EPI_ISL_7778778 | A/common_eider/Norway/FU453_21VIR7634-3/2021 |
| EPI_ISL_17414585 | A/avian/Nigeria/120_22VIR3286-62/2022 | EPI_ISL_292225 | A/canada_goose/England/AV58_18OPpoolEP1/2018 | EPI_ISL_7778779 | A/common_eider/Norway/FU458_21VIR7634-4/2021 |
| EPI_ISL_17414594 | A/avian/Nigeria/737_22VIR3286-25/2021 | EPI_ISL_292240 | A/Bar-headed_Goose/Qinghai/p23/2016 | EPI_ISL_7778780 | A/common_eider/Norway/FU474_21VIR7634-5/2021 |
| EPI_ISL_17414608 | A/avian/Nigeria/VRD-21-140_21VIR7423-19/2021 | EPI_ISL_292330 | A/Bar-headed_Goose/Qinghai/a26/2016 | EPI_ISL_79686 | A/Hunan/1/2009 |
| EPI_ISL_17414619 | A/chicken/Nigeria/030_22VIR3286-47/2022 | EPI_ISL_292334 | A/Bar-headed_Goose/Qinghai/B655/2017 | EPI_ISL_79693 | A/environment/Guizhou/2/2009 |
| EPI_ISL_17414622 | A/chicken/Nigeria/040_22VIR3286-50/2022 | EPI_ISL_2932608 | A/chicken/Kazakhstan/220-B-2-H5N8-4/2020 | EPI_ISL_79695 | A/environment/Guizhou/7/2009 |
| EPI_ISL_17414641 | A/chicken/Nigeria/648_22VIR3286-5/2021 | EPI_ISL_2932616 | A/chicken/Kazakhstan/12-20-B-Talg-45/2020 | EPI_ISL_7983813 | A/Hangzhou/01/2021 |
| EPI_ISL_17414655 | A/chicken/Nigeria/VRD-21-212_21VIR7423-5/2021 | EPI_ISL_293952 | A/duck/France/160927/2016 | EPI_ISL_7996371 | A/turkey/Netherlands/21040980-001005/2021 |
| EPI_ISL_17414658 | A/chicken/Nigeria/VRD-21-361_21VIR7423-34/2021 | EPI_ISL_293983 | A/guineafowl/France/160009/2016 | EPI_ISL_80641 | A/chicken/Vietnam/NCVD-093/2008 |
| EPI_ISL_17424629 | A/black_vulture/South_Carolina/W22-1080A/2022 | EPI_ISL_293984 | A/duck/France/150206/2015 | EPI_ISL_813979 | A/peregrine_falcon/Ireland/20VIR7872-1/2020 |
| EPI_ISL_17424644 | A/eagle/South_Carolina/W23-142A/2023 | EPI_ISL_293985 | A/guineafowl/France/150223/2015 | EPI_ISL_8215689 | A/Bar-headed_Goose/Tibet/XZ1131/2021 |
| EPI_ISL_174380 | A/broiler_duck/Korea/H1731/2014 | EPI_ISL_293987 | A/chicken/France/160102/2016 | EPI_ISL_833248 | A/Muscovy_duck/China/FJFZ21/H5N6/2020 |
| EPI_ISL_17514129 | A/chicken/Austria/23003433-002/2023 | EPI_ISL_293992 | A/chicken/France/160001/2016 | EPI_ISL_8377254 | A/egret/France/21P013418/2021 |
| EPI_ISL_17564760 | A/chicken/Wales/057026/2023 | EPI_ISL_293997 | A/chicken/France/160013/2016 | EPI_ISL_8515483 | A/great_egret/Czech_Republic/23609/2021 |
| EPI_ISL_17584744 | A/duck/France/22010/2022 | EPI_ISL_294080 | A/goose/France/150294/2015 | EPI_ISL_8518268 | A/Chicken/Hangzhou/E1149/2021 |
| EPI_ISL_17599531 | A/Corvus_monedula/Belgium/02027_0007/2023 | EPI_ISL_294125 | A/duck/France/150254/2015 | EPI_ISL_8768947 | A/turkey/Tyumen/15-3V/2021 |
| EPI_ISL_1760445 | A/Hong_Kong/213/2003 | EPI_ISL_294600 | A/Von_Schrenck_s_bittern/Jiangxi/Y9/2014 | EPI_ISL_8768953 | A/turkey/Tyumen/15-14V/2021 |
| EPI_ISL_1760448 | A/common_teal/Shaanxi/SXY1-1/2020 | EPI_ISL_294758 | A/mallard/France/150018/2015 | EPI_ISL_8769017 | A/chicken/Tyumen/47-66V/2021 |
| EPI_ISL_1760450 | A/whooper_swan/Shaanxi/SXY2-1/2020 | EPI_ISL_294761 | A/duck/France/150213/2015 | EPI_ISL_8769018 | A/chicken/Tyumen/47-79V/2021 |
| EPI_ISL_1760451 | A/whooper_swan/Shanxi/SX16/2020 | EPI_ISL_294770 | A/duck/France/150234/2015 | EPI_ISL_8769034 | A/chicken/Tyumen/81-97V/2021 |
| EPI_ISL_17606815 | A/mandarin_duck/Korea/WA622/2021 | EPI_ISL_294778 | A/duck/France/160056/2016 | EPI_ISL_9009296 | A/chicken/Rostov-on-Don/159-1V/2021 |
| EPI_ISL_17606817 | A/mallard/Korea/WA44/2022 | EPI_ISL_294779 | A/duck/France/160051/2016 | EPI_ISL_9009483 | A/chicken/Nghe_An/7007VTC/2020 |
| EPI_ISL_17606823 | A/chicken/Korea/H559/2021 | EPI_ISL_295027 | A/chicken/Kostroma/1718/2017 | EPI_ISL_9009515 | A/chicken/Thanh_Hoa/1351VTC/2021 |
| EPI_ISL_17606828 | A/duck/Korea/H608/2021 | EPI_ISL_295505 | A/Gadwall/Ningxia/485-31/2015 | EPI_ISL_9012457 | A/chicken/England/053052/2021 |
| EPI_ISL_17606829 | A/duck/Korea/H609/2021 | EPI_ISL_295752 | A/black-headed_gull/Hyogo/2801E009/2017 | EPI_ISL_9012618 | A/chicken/Wales/053969/2021 |
| EPI_ISL_17606831 | A/duck/Korea/H621/2021 | EPI_ISL_295818 | A/muscovy_duck/Vietnam/HU7-17/2017 | EPI_ISL_9012696 | A/chicken/Scotland/054477/2021 |
| EPI_ISL_17606833 | A/quail/Korea/H624/2021 | EPI_ISL_296405 | A/swine/Iowa/A02218427/2017 | EPI_ISL_9029965 | A/Greylag_goose/England/054503/2021 |
| EPI_ISL_17606834 | A/chicken/Korea/H625/2021 | EPI_ISL_300662 | A/tundra_swan/Niigata/5112006/2016 | EPI_ISL_9250718 | A/Gallus_gallus/Belgium/11372_0001/2021 |
| EPI_ISL_17606836 | A/duck/Korea/H640/2021 | EPI_ISL_300702 | A/chicken/Poland/101/2017 | EPI_ISL_9261742 | A/Great_black-backed_gull/1/2022 |
| EPI_ISL_17606837 | A/duck/Korea/H649/2021 | EPI_ISL_303837 | A/grey-headed_gull/Uganda/MUWRP-538/2017 | EPI_ISL_9324 | A/Chicken/Yunnan/447/05 |
| EPI_ISL_17606838 | A/duck/Korea/H13/2022 | EPI_ISL_304404 | A/Fujian-Sanyuan/21099/2017 | EPI_ISL_9325 | A/Chicken/Yunnan/493/05 |
| EPI_ISL_17606839 | A/duck/Korea/H38/2022 | EPI_ISL_304958 | A/chicken/Vietnam/QuangBinh/BD1113/2017 | EPI_ISL_9328 | A/Goose/Shantou/1621/05 |
| EPI_ISL_17606841 | A/chicken/Korea/H57/2022 | EPI_ISL_305453 | A/chicken/Germany-SH/AR163-L02542/2018 | EPI_ISL_9377021 | A/goose/France/21P014207/2021 |
| EPI_ISL_17606842 | A/chicken/Korea/H58/2022 | EPI_ISL_306912 | A/Chicken/Riyadh/AI6/2017 | EPI_ISL_94329 | A/duck/Eastern_China/008/2008 |
| EPI_ISL_17606845 | A/duck/Korea/H87/2022 | EPI_ISL_3090099 | A/Sichuan/06681/2021 | EPI_ISL_9572656 | A/chicken/Nigeria/VRD-19-023_19RS1081-1/2019 |
| EPI_ISL_17606846 | A/chicken/Korea/H88/2022 | EPI_ISL_309196 | A/Buteo_buteo/Belgium/3022/2017 | EPI_ISL_9572672 | A/chicken/Nigeria/VRD-18-NS10_18RS1971-29/2018 |
| EPI_ISL_17606848 | A/chicken/Korea/H91/2022 | EPI_ISL_3102073 | A/turkey/Poland/H1184_21RS1385-14/2021 | EPI_ISL_9572739 | A/Muscovy_duck/Vietnam/HN6610/2020 |
| EPI_ISL_17606849 | A/Korean_native_chicken/Korea/H97/2022 | EPI_ISL_3102078 | A/mute_swan/Poland/MB396_21RS1385-19/2021 | EPI_ISL_9572741 | A/duck/Vietnam/HN6611/2020 |
| EPI_ISL_17606853 | A/chicken/Korea/H129/2022 | EPI_ISL_3144489 | RG-A/chicken/Zhejiang/HJ/2007 | EPI_ISL_9572754 | A/Muscovy_duck/Vietnam/HN6606/2020 |
| EPI_ISL_17606855 | A/duck/Korea/H138/2022 | EPI_ISL_314984 | A/Vietnam/1194/2004 | EPI_ISL_9572759 | A/Muscovy_duck/Vietnam/HN6607/2020 |
| EPI_ISL_17606856 | A/quail/Korea/H139/2022 | EPI_ISL_315237 | A/Chicken/Xuzhou/470/2017 | EPI_ISL_9572763 | A/duck/Vietnam/HN6423/2020 |
| EPI_ISL_17606857 | A/chicken/Korea/H141/2022 | EPI_ISL_316551 | A/chicken/Italy/17VIR9107/2017 | EPI_ISL_9572770 | A/Muscovy_duck/Vietnam/HN6608/2020 |
| EPI_ISL_17606859 | A/chicken/Korea/H154-1/2022 | EPI_ISL_316553 | A/chicken/Italy/17VIR9113/2017 | EPI_ISL_9572776 | A/duck/Vietnam/HN6424/2020 |
| EPI_ISL_17606862 | A/duck/Korea/H200/2022 | EPI_ISL_320607 | A/chicken/Kursk/284/2018 | EPI_ISL_9572797 | A/duck/Vietnam/HN6036/2019 |
| EPI_ISL_17606864 | A/chicken/Korea/H384/2022 | EPI_ISL_322179 | A/domestic_duck/Germany-MV/AR613-L02727/2018 | EPI_ISL_9572815 | A/duck/Vietnam/HN6038/2019 |
| EPI_ISL_17638141 | A/chicken/Hokkaido/HU-B102/2023 | EPI_ISL_328195 | A/chicken/China/GZ1063/2014 | EPI_ISL_9594290 | A/chicken/Egypt/A19670/2021 |
| EPI_ISL_17672077 | A/black-headed_gull/Luxembourg/23111454/2023 | EPI_ISL_328955 | A/mallard_duck/Netherlands/52/2015 | EPI_ISL_9603920 | A/mute_swan/Czech_Republic/22380/2021 |
| EPI_ISL_17710021 | A/Black-Headed_Gull/England/059292/2023 | EPI_ISL_3290184 | A/Canada_Goose/Sweden/SVA210330SZ0426/FB001307/E-2021 | EPI_ISL_9616212 | A/red_knot/Netherlands/22000409-002/2022 |
| EPI_ISL_17710046 | A/Black-Headed_Gull/England/125703/2023 | EPI_ISL_331119 | A/duck/Bangladesh/17D1012/2018 | EPI_ISL_964 | A/Hong_Kong/483/1997 |
| EPI_ISL_17716062 | A/domestic_duck/Austria/23011278-027/2023 | EPI_ISL_332439 | A/Perigrine_falcon/Netherlands/18003274-001/2018 | EPI_ISL_96896 | A/Hubei/1/2010 |
| EPI_ISL_17728623 | A/duck/China/Q35-M55/2017 | EPI_ISL_332441 | A/Mallard/Netherlands/18012508-017/2018 | EPI_ISL_977582 | A/duck/Northern_China/ZGL/2020 |
| EPI_ISL_17728625 | A/goose/China/Q99-PB237/2017 | EPI_ISL_333367 | A/Environment/Jiangxi/47054/2016 | EPI_ISL_977599 | A/duck/Northern_China/LSP/2020 |
| EPI_ISL_17728650 | A/goose/China/Q183-PB120/2017 | EPI_ISL_3336 | A/Chicken/HongKong/YU562/01 | EPI_ISL_985182 | A/chicken/Korea/H390/2020 |
| EPI_ISL_17728655 | A/goose/China/Q183-M15/2017 | EPI_ISL_333615 | A/gadwall/Chany/893/2018 | EPI_ISL_985192 | A/chicken/Korea/H441/2020 |
| EPI_ISL_177584 | A/domestic_duck/Hungary/7341/2015 | EPI_ISL_335273 | A/Jungle_crow/Hyogo/2803E023T/2018 | EPI_ISL_985193 | A/chicken/Korea/H450/2020 |
| EPI_ISL_17760672 | A/chicken/Hebei/CK05/2019 | EPI_ISL_335446 | A/duck/Korea/H192/2018 | EPI_ISL_985195 | A/chicken/Korea/H470/2020 |
| EPI_ISL_17760697 | A/duck/China/10-14/2022 | EPI_ISL_337274 | A/Guangdong/18SF020/2018 | EPI_ISL_98855 | A/Indonesia/NIHRD11771/2011 |
| EPI_ISL_17760699 | A/duck/China/10-26/2022 | EPI_ISL_337275 | A/Jiangsu/32888/2018 | EPI_ISL_9895 | A/chicken/Fujian/1042/2005 |
| EPI_ISL_17760704 | A/duck/China/L41/2022 | EPI_ISL_337280 | A/Guangxi/13486/2017 | EPI_ISL_9899 | A/goose/Guangxi/914/2004 |
| EPI_ISL_17760709 | A/duck/China/C1/2022 | EPI_ISL_340788 | A/Env/Guangdong/zhanjiang/C17277346/2017-12-05 | EPI_ISL_9902 | A/duck/Guangxi/1311/2004 |
| EPI_ISL_17760710 | A/duck/China/C2/2022 | EPI_ISL_340789 | A/Duck/Guangdong/PO17281256/MZH/2017-8-21 | EPI_ISL_9910 | A/goose/Guangxi/2383/2004 |
| EPI_ISL_17760712 | A/duck/China/9-11/2022 | EPI_ISL_340791 | A/Env/Guangdong/C172811415/MZH/2017-10-16 | EPI_ISL_9914 | A/chicken/Guangxi/2461/2004 |
| EPI_ISL_17760714 | A/duck/China/9-18/2022 | EPI_ISL_340792 | A/Duck/Guangdong/PO17281388/MZH/2017-10-24 | EPI_ISL_9921 | A/duck/Hunan/127/2005 |
| EPI_ISL_177649 | A/eurasian_wigeon/Netherlands/1/2015 | EPI_ISL_340793 | A/Env/Guangdong/C172811414/MZH/2017-10-16 | EPI_ISL_9929 | A/chicken/Hunan/999/2005 |

**References**

1. Khare S, Gurry C, Freitas L, Schultz MB, Bach G, Diallo A, Akite N, Ho J, Lee RT, Yeo W, Curation Team GC, Maurer-Stroh S. 2021. GISAID's Role in Pandemic Response. China CDC Wkly 3:1049-1051.

2. Camacho C, Coulouris G, Avagyan V, Ma N, Papadopoulos J, Bealer K, Madden TL. 2009. BLAST+: architecture and applications. BMC Bioinformatics 10:421.

3. Adlhoch C, Fusaro A, Gonzales JL, Kuiken T, Marangon S, Niqueux É, Staubach C, Terregino C, Aznar I, Guajardo IM, Lima E, Baldinelli F. 2021. Avian influenza overview February – May 2021. EFSA journal 19:e06951-n/a.

4. Adlhoch C, Fusaro A, Gonzales JL, Kuiken T, Mirinavičiūtė G, Niqueux É, Staubach C, Terregino C, Baldinelli F, Rusinà A, Kohnle L. 2023. Avian influenza overview June–September 2023. EFSA journal 21.

5. Cruz CD, Icochea ME, Espejo V, Troncos G, Castro-Sanguinetti GR, Schilling MA, Tinoco Y. 2023. Highly Pathogenic Avian Influenza A from Wild Birds, Poultry, and Mammals, Peru. Emerging infectious diseases 29:2572-2576.

6. Suttie A, Deng YM, Greenhill AR, Dussart P, Horwood PF, Karlsson EA. 2019. Inventory of molecular markers affecting biological characteristics of avian influenza A viruses. Virus Genes 55:739-768.

7. Zhu W, Li X, Dong J, Bo H, Liu J, Yang J, Zhang Y, Wei H, Huang W, Zhao X, Chen T, Yang J, Li Z, Zeng X, Li C, Tang J, Xin L, Gao R, Liu L, Tan M, Shu Y, Yang L, Wang D. 2022. Epidemiologic, Clinical, and Genetic Characteristics of Human Infections with Influenza A(H5N6) Viruses, China. Emerging infectious diseases 28:1332-1344.
